# Insights into SCP/TAPS Proteins of Liver Flukes Based on Large-Scale Bioinformatic Analyses of Sequence Datasets

**DOI:** 10.1371/journal.pone.0031164

**Published:** 2012-02-22

**Authors:** Cinzia Cantacessi, Andreas Hofmann, Neil D. Young, Ursula Broder, Ross S. Hall, Alex Loukas, Robin B. Gasser

**Affiliations:** 1 Department of Veterinary Science, The University of Melbourne, Parkville, Victoria, Australia; 2 Eskitis Institute for Cell and Molecular Therapies, Griffith University, Brisbane, Queensland, Australia; 3 Queensland Tropical Health Alliance, James Cook University, Smithfield, Queensland, Australia; Seoul National University College of Medicine, Republic of Korea

## Abstract

**Background:**

SCP/TAPS proteins of parasitic helminths have been proposed to play key roles in fundamental biological processes linked to the invasion of and establishment in their mammalian host animals, such as the transition from free-living to parasitic stages and the modulation of host immune responses. Despite the evidence that SCP/TAPS proteins of parasitic nematodes are involved in host-parasite interactions, there is a paucity of information on this protein family for parasitic trematodes of socio-economic importance.

**Methodology/Principal Findings:**

We conducted the first large-scale study of SCP/TAPS proteins of a range of parasitic trematodes of both human and veterinary importance (including the liver flukes *Clonorchis sinensis*, *Opisthorchis viverrini*, *Fasciola hepatica* and *F. gigantica* as well as the blood flukes *Schistosoma mansoni*, *S. japonicum* and *S. haematobium*). We mined all current transcriptomic and/or genomic sequence datasets from public databases, predicted secondary structures of full-length protein sequences, undertook systematic phylogenetic analyses and investigated the differential transcription of SCP/TAPS genes in *O. viverrini* and *F. hepatica*, with an emphasis on those that are up-regulated in the developmental stages infecting the mammalian host.

**Conclusions:**

This work, which sheds new light on SCP/TAPS proteins, guides future structural and functional explorations of key SCP/TAPS molecules associated with diseases caused by flatworms. Future fundamental investigations of these molecules in parasites and the integration of structural and functional data could lead to new approaches for the control of parasitic diseases.

## Introduction

The SCP/Tpx-1/Ag5/PR-1/Sc7 (SCP/TAPS) protein family (Pfam accession number no. PF00188; InterPro accession number IPR014044) includes a range of structurally related proteins found in a wide range of eukaryotes [1] and characterized by the presence of SCP-extracellular domains (a single- or double-domain SCP/TAPS) which act as Ca^2+^-chelators in various signalling processes [2]. SCP/TAPS proteins include rodent sperm-coating glycoproteins (or acidic glycoproteins, proposed to be involved in sperm maturation during passage through the epididymis) [3], mammalian testis-specific protein (Tpx-1) [4], glioma pathogenesis-related protein [5]–[7], venom allergen 5 from vespid wasps and the venom allergen 3 from fire ants, which mediate allergic reactions to the bites by some insects of the order Hymenoptera [8] as well as plant pathogenesis proteins (PRPs) of the PR-1 “subfamily” which are synthesized in response to infections with pathogens or other stress-inducing factors [9]. For parasitic helminths, SCP/TAPS proteins are common in nematodes of the orders Spirurida, Ascaridida, Tylenchida, Rhabditida and Strongylida [10]. Within the latter order, members of the SCP/TAPS protein family have been well studied in the canine hookworm, *Ancylostoma caninum*. Based on the observation that these proteins are abundant in the excretory/secretory (ES) products of the serum-activated third-stage larvae (L3s), they have been designated as ‘*Ancylostoma*-secreted proteins’ or ‘activation-associated secreted proteins’ ( = ASPs; [11], [12]). In hookworms, SCP/TAPS proteins are thought to play an important role in the transition from the free-living to the parasitic stage of the L3s during the invasion of and the migration through the host's tissues [11]–[15], as well as key role/s in the modulation of the host's immune response [14], [16]. In addition, because of its immunogenic properties, one SCP/TAPS protein (called *Na*-ASP-2) is currently under investigation as a vaccine candidate against the disease ( = necatoriasis) caused by the human hookworm *Necator americanus* [17]–[20].

Despite the fundamental roles that nematode SCP/TAPS proteins are proposed to play in the host-parasite interplay [1], [11]–[15], knowledge of SCP/TAPS homologues/orthologues in parasitic trematodes ( = flukes) is scant. In a single study, Chalmers et al. [21] identified genes encoding SCP/TAPS proteins (designated SmVALs) of *Schistosoma mansoni* (a blood fluke of humans) by mining of available expressed sequence tag (EST) datasets [21], investigated levels of transcription of corresponding mRNAs in different developmental stages of this parasite, performed extensive analyses of the predicted amino acid sequences by homology modelling, and inferred phylogenetic relationships [21]. Based on the results from this study, the authors proposed a role for SmVALs in processes linked to the invasion of the human host by *S. mansoni* [21]. This hypothesis requires testing. In addition, elucidating the structure/s and function/s of these molecules in *S. mansoni* and other socioeconomically important parasitic trematodes could provide an avenue for the design of new approaches for their control.

Recently, advances in next-generation sequencing (NGS) and bioinformatics [22]–[25] have allowed large-scale explorations of the transcriptomes and/or genomes of a range of parasitic trematodes, including the carcinogenic opisthorchiids *Clonorchis sinensis* and *Opisthorchis viverrini* [26], the fasciolids *Fasciola hepatica* [27] and *F. gigantica* [28] (liver flukes) as well as *Schistosoma mansoni* and *S. japonicum* [29], [30] (blood flukes). The sequence data generated in these studies, available for download from public databases (e.g., http://www.gasserlab.org/ and http://www.genedb.org/), represent an excellent resource for studies of SCP/TAPS proteins in parasitic trematodes. Utilizing current datasets, we conduct herein the first large-scale analysis of SCP/TAPS proteins in a range of parasitic trematodes of both human and veterinary health importance; infer relationships between/among trematode SCP/TAPS based on predictions of secondary structures of protein sequences; and investigate differences in the transcription of genes encoding SCP/TAPS between the juvenile and adult stages of *O. viverrini* and *F. hepatica*.

## Materials and Methods

### Sequence datasets, and identification and analyses of SCP/TAPS homologues/orthologues

The sequence data obtained from public sequence databases (i.e. http://www.gasserlab.org/ and http://www.genedb.org/) [26]–[31] and analysed herein included predicted peptide inferred from (i) the transcriptome [generated by 454 sequencing of normalized complementary DNA (cDNA) libraries] of the adult stage of *C. sinensis* (*n* = 50,769 predicted peptides) [Sequence Read Archive (SRA) accession number: SRA012272]; (ii) the transcriptomes [generated by Illumina sequencing of non-normalized cDNA libraries] of adult *F. gigantica* (*n* = 30,525) (SRA024257) and of both adult and juvenile stages of *O. viverrini* (*n* = 25,172) and *F. hepatica* (*n* = 19,669) (http://www.gasserlab.org/); (iii) the genome sequences of *S. mansoni* (*n* = 13,174 peptides), *S. japonicum* (*n* = 13,469) and *S. haematobium* (*n* = 13,073). The algorithms BLASTp [32] and InterProScan [33] were used to identify single- and double-domain SCP/TAPS (predicted) in each of the transcriptomic and genomic datasets based on sequence homology (e-value cut-off: 10^−5^) with known eukaryotic SCP/TAPS proteins (cf. [1]), and on the presence of one or more SCP-extracellular domains (Pfam: PF00188; InterPro: IPR014044), respectively. Signal peptides were also predicted using the program SignalP 3.0, employing both the neural network and hidden Markov Models [34]. Putative excreted/secreted SCP/TAPS proteins were identified based on the presence of a signal peptide and sequence homology to one or more known ES proteins listed in the Secreted Protein (http://spd.cbi.pku.edu.cn/; [35]) and the Signal Peptide (http://proline.bic.nus.edu.sg/spdb/index.html; [36]) databases.

### Prediction of the secondary structures of trematode SCP/TAPS and homology modelling

Structure-based sequence alignments of both single- and double-domain SCP/TAPS were generated manually, guided by secondary structure elements predicted using PSIPRED software [37]. Individual structure-based alignments of amino acid sequences (>120 amino acids in length) were subjected to analysis by Bayesian inference (BI) using the program MrBayes v.3.1.2 [38] and verified by Neighbour Joining (NJ) analysis using the MEGA software [39]. Each BI analysis was conducted for 1,000,000 generations (ngen = 1,000,000), with every 100-th tree being saved, using the following parameters: rates = gamma, aamodelpr = mixed, and the other parameters left at the default settings. Tree and branch lengths were measured employing the parameter ‘sumt burnin = 1000’; an unrooted, consensus tree was constructed, with ‘contype = halfcompat’ nodal support being determined using consensus posterior probabilities and displayed employing the program TreeView v.1.6.6 [40]. For selected single-domain SCP/TAPS proteins, homologues with known three-dimensional structures were identified using the protein-fold recognition software pGenTHREADER [41] and selected as templates for comparative modelling using MODELLER [42]. Twenty independent models were generated, and the model with the lowest energy was selected, its geometry analysed using PROCHECK [43] and then inspected visually with PyMOL [44].

### Assessment of levels of transcription of genes encoding SCP/TAPS in selected liver flukes

The raw sequence reads derived from each of the non-normalized cDNA libraries from adult and juvenile *O. viverrini* and *F. hepatica* were mapped to the longest contigs representing individual SCP/TAPS proteins using the program SOAP2 [45]. Briefly, raw sequence reads were aligned to the non-redundant transcriptomic data, such that each raw sequence read was uniquely mapped (i.e. to a unique transcript). Reads that mapped to more than one transcript (designated ‘multi-reads’) were randomly assigned to a unique transcript, such that they were recorded only once. To provide a relative assessment of transcript abundance, the number of raw reads that mapped to each sequence was normalized for length (i.e. reads per kilobase per million reads, RPKM) [46].

### Interaction networking

An established method [47] was used for probabilistic functional genetic networking among *Mus musculus* gene homologues/orthologues of molecules encoding SCP/TAPS proteins of parasitic trematodes using the recommended, stringent cut-off value of 4.6. The predicted networks resulting from the analyses were saved in a graphic display file (gdf) format, examined using the graph exploration system available at http://graphexploration.cond.org/ (http://www.geneorienteer.org/; [47]).

## Results and Discussion

### SCP/TAPS proteins of parasitic trematodes

A total number of 151 peptides with high homology (e-value cut-off: 10^−5^) to known eukaryotic SCP/TAPS were predicted from all of the genomic and/or transcriptomic sequence datasets available for trematodes (Tables S1 and S2). These datasets provide a solid resource for future structural and functional investigations of the SCP/TAPS protein family of trematodes and other parasitic helminths. Of the sequence data included here, the complement of protein-coding genes of *S. mansoni* comprises the largest number of predicted SCP/TAPS proteins reported to date (*n* = 39 and 2 single- and double-domain SCP/TAPS, respectively) (Tables S1 and S2). One of these amino acid sequences (i.e. Smp_131370) had not been predicted from the *S. mansoni* EST datasets analysed previously [21], thus representing a novel record. The large number of transcripts encoding distinct SCP/TAPS proteins in *S. mansoni* is in contrast to the number of SCP/TAPS-encoding genes inferred from the genomic sequence data from the other two *Schistosoma* species analysed herein (*n* = 17 and 25 for *S. japonicum* and *S. haematobium*, respectively; cf. Tables S1 and S2). The likely explanation for this result is technical and appears to relate to the fact that, in the assemblies of the *S. japonicum* and *S. haematobium* genomes, smaller proportions of genome sequence are contained within large, contiguous sequences (‘scaffolds’) [30], [31], thus leading to the fragmentation of predicted open reading frames (ORFs) and, in turn, to an underestimation of the number of protein-coding genes. In the future, bridging the gaps between scaffolds representing these draft genomes (by, for instance, performing re-assembly of Illumina reads to close gaps between adjacent contigs within scaffolds; [48]) should allow the unequivocal identification of the complete sets of SCP/TAPS protein-coding genes in these blood flukes and will pave the way for comparative studies. In addition, it will provide a basis for in-depth analyses of amino acid sequence features, such as patterns of cysteine residues and presence/absence of signal peptides, thus assisting future structural and functional analyses of members of the SCP/TAPS protein family in parasitic helminths.

In the present study, a total number of 42 (27%) SCP/TAPS amino acid sequences were predicted to contain an N-terminal signal peptide (Tables S1 and S2); in particular, amongst the sequence data analysed herein, the *S. mansoni* set of predicted proteins included the largest number of SCP/TAPS with a signal peptide indicative of secretion (*n* = 17; Tables S1 and S2). Conversely, none of the SCP/TAPS amino acid sequences predicted from the transcriptome of *F. gigantica* contained a secretory signal peptide (Tables S1 and S2), despite unpublished evidence of one SCP/TAPS containing a signal peptide in this trematode (GenBank accession number FN379399). These findings support the existence of two types of eukaryotic SCP/TAPS proteins, one of which lacks a signal peptide and is localized within the cellular compartment, in association with the Golgi endoplasmic reticulum (e.g., the Golgi-associated PR-1-related protein [GAPR-1] of vertebrates; [49]–[51]), and the other which contains a signal peptide and is localized in the extracellular compartment (e.g., *Ac*-ASP-2 of *A. caninum*; [12]). The detection of SCP/TAPS amino acid sequences that lack a predicted signal peptide in all of the trematode sequence datasets contrasts the situation for parasitic nematodes (including hookworms and filarioids) whose SCP/TAPS proteins usually possess a signal peptide and are abundant in the ES products (e.g., [1], [12], [15], [52]–[55]). To date, SCP/TAPS proteins have been identified in the ES products of various species and different developmental stages of parasitic trematodes, including *S. mansoni* and *S. japonicum* eggs [56], [57] and *O. viverrini* adults [58]. In particular, protein *Sj*-VAL-1 was isolated from the ES products of eggs of *S. japonicum* and shown to evoke a specific Th2-type immune response when inoculated into naïve mice [56], whereas other SCP/TAPS members have been isolated from ES products from *S. mansoni* miracidia and cercariae during their transition to sporocysts [59] and schistosomules [60], [61], respectively. These findings raise questions as to the roles that SCP/TAPS play in the infection process in both the vertebrate and molluscan hosts. In another study, SCP/TAPS proteins were not detected in ES products from juvenile or adult *F. hepatica* [62]. The structural and functional differences between secreted and non-secreted SCP/TAPS proteins remain unclear [21] and warrant detailed investigations. Both secreted and non-secreted SCP/TAPS are characterized by the presence of a highly conserved SCP-domain [63]. Although conserved domains are known to play key roles in determining protein function, protein-protein interactions, DNA binding and enzyme activity [64], structural analyses of the complete amino acid sequence of proteins are essential to assist in-depth investigations of function [65].

### Structural classification of trematode SCP/TAPS

**Figure 1:**
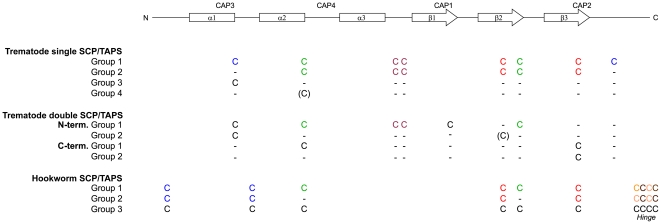
Schematic of the topology of the SCP-fold, location of the *c*ysteine-rich secretory proteins, *a*ntigen 5, and *p*athogenesis-related 1 proteins (CAP) motifs and approximate position of conserved cysteine residues in the primary structure. The proposed classification of groups of trematode SCP/TAPS proteins is supported by the occurrence of conserved cysteine residues. The grouping of hookworm SCP/TAPS proteins has been reported recently [63]. For the hookworm SCP/TAPS proteins, the cysteine connectivity in the intra-molecular disulphide bonds is known from experimental three-dimensional structures and shown by colour-mapping. The likely cysteine connectivity for trematode SCP/TAPS is hypothetical and based on the modelling in this study.

**Figure 2:**
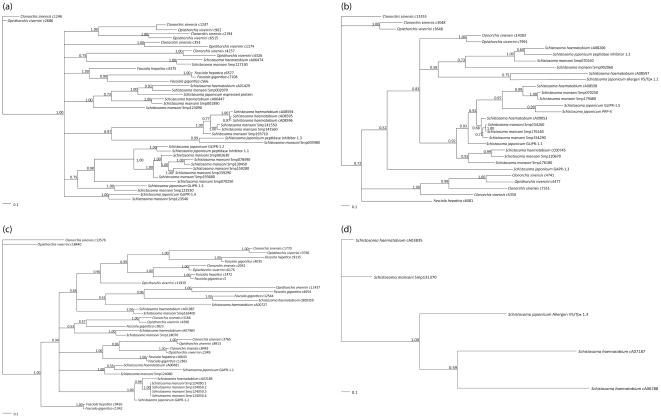
The phylogenetic relationships of single-domain SCP/TAPS proteins (>120 amino acids in length) predicted from the transcriptomes of *Clonorchis sinensis*, *Opisthorchis viverrini*, *Fasciola hepatica* and *F. gigantica* (liver flukes) and the genomes of *Schistosoma mansoni*, *S. japonicum* and *S. haematobium* (blood flukes) based on Bayesian inference. Group 1 (a); group 2 (b); group 3 (c); group 4 (d). The posterior probability supporting each clade is indicated. The corresponding phylogenetic reconstructions conducted using Neighbour Joining analyses of single-domain SCP/TAPS proteins are available from the primary author upon request.

In the present study, a key criterion for the classification of trematode SCP/TAPS proteins was the presence of cysteine residues at particular sequence positions. Three conserved disulphide bonds, which stabilize the fold of the core, were defined as a hallmark-feature of the SCP domain. For single-domain SCP/TAPS proteins, amino acid sequence alignments, guided by predictions of their secondary structures and sequence similarity, allowed the definition of (at least) four individual groups (Table S1; Figures 1 and 2a–d; Figure S1), characterized by: (i) the presence of all conserved secondary structure elements of the SCP domain, an N-terminal α-helix in most sequences and eight conserved cysteine residues (group 1); (ii) presence of all conserved secondary structure elements of the SCP domain, an N-terminal α-helix in many sequences, a cysteine-rich N-terminal region and the absence of conserved cysteine residues after α1 and β3 (group 2); (iii) presence of all conserved secondary structure elements of the SCP domain and of one conserved cysteine residue after α1, and the absence of an N-terminal α-helix (group 3); or (iv) distribution of secondary structure elements, similar to those of groups 1–3, and the absence of conserved cysteine residues (group 4). Within group 3, *F. gigantica* c4654, *F. gigantica* c12544, *O. viverrini* c3766, *C. sinensis* c8455 and *O. viverrini* c2349 did not possess the cysteine residue at α1, characteristic for this group; however, the similarity between their predicted secondary structures and those of other proteins within group 3 led to their inclusion within this group. The SCP/TAPS groups differ markedly in the conservation of cysteine residues; while the conserved intra-molecular disulphide bonds are present in proteins of groups 1 and 2, they are absent from those of groups 3 and 4. Thus far, the only known example of a non-disulphide-stabilised SCP-fold is GAPR-1 [66]. Of the 148 SCP/TAPS amino acid sequences predicted, eleven represented double-domain SCP/TAPS proteins. However, the structure-based amino acid sequence alignment revealed that the C-terminal moiety of two of these proteins, namely *S. haematobium* cA00818 and cA08278, possessed a non-SCP/TAPS fold. Of the remaining nine sequences, *S. haematobium* cA07851 represented group 1, and the other eight sequences were classified as group 2, based on the conservation of cysteine residues (Table S2; Figure 1; Figure S2). Previously, structure-based sequence alignments of SCP/TAPS proteins of nematodes had led to their categorization into three (structural) groups [63]. Based on comparisons of the positions of the conserved cysteine residues between nematode and trematode SCP/TAPS proteins, disulphide bridges can be inferred that are crucial for the fold-stability (α2-β2 and β2-β3) and for the tolerability of variations of the molecular constituents of the SCP-fold. In particular, up to four conserved cysteine residues, including those linking α2-β2 and β2- β3, in the amino acid sequences of single-domain SCP/TAPS belonging to group 3 and 4, are mutated.

**Figure 3:**
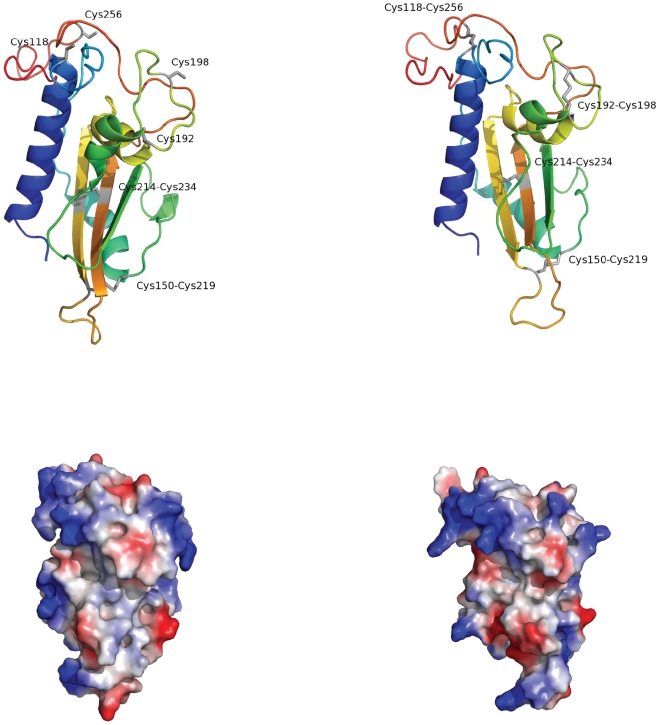
Homology models for the molecule c962 from *Opisthorchis viverrini*. Top row: the models are rendered in cartoon representation with cysteine side chains shown as bars in grey. The colour mapping ramps from blue (N-terminal end) to red (C-terminal end). Bottom row: surface representation of the models is in the same orientation as in the top row. The electrostatic surface potential is mapped by colour (blue: positive charge; red: negative charge). The left panel shows a homology model using *Na*-ASP-2 (PDB code: 1u53) as a template without restraint. For the model shown in the right panel, two restraints were applied to force disulphide bonds between the remaining cysteine residues. The comparison of both models highlights the significant differences in conformation and shape resulting from different template restraints applied. The images were generated using PyMOL [http://www.pymol.org/].

At the molecular level, SCP/TAPS proteins adopt the fold of an α-β-α sandwich, similar to the plant PR-1 protein P14a [2] and the hookworm protein *Na*-ASP-2 [67]. Variable extensions are linked to the C-terminus of the SCP-extracellular domain and include the LCCL ( = Limulus clotting factor C, Coch-5b2, and Lgl1), the C-type lectin, the ion channel regulator [66] and an additional SCP-extracellular domain. The abundance of the SCP/TAPS fold and its extension, by means of a variable set of protein domains with distinct functions, suggest a “vehicle-payload” model for these proteins [63], whereby the C-terminally linked domain is delivered by the SCP-domain to sites of action. The SCP-fold could also be hypothesized to be involved in binding, recognition or enzymatic activities; however, further studies are required to support this hypothesis. In the future, experimental studies aimed at defining the structure-function relationships of the SCP-fold will provide a solid basis for determining its precise molecular activities. Indeed, given the magnitude of variation in the atomic structure of the SCP-fold, three-dimensional models obtained by comparative modelling need to be treated with caution. To illustrate this point, we generated and critically appraised the homology model for a group 1, single-domain SCP/TAPS protein (designated c962) from *O. viverrini*. For this target, the structure of *Na*-ASP-2 (PDB code 1u53) was identified as the best template for comparative modelling. An analysis of the predicted structure revealed that the cysteine residues that are conserved between the trematode group 1 and the hookworm group 1 SCP/TAPS proteins are engaged in disulphide bonds, due to the restraints provided by the chosen template. The remaining, even number of cysteine residues may also engage in intra-molecular disulphide bonds, and their positioning within the primary structure promotes this hypothesis. Due to the restraints by the chosen template, the homology model for the group 1 proteins of trematodes shows that these additional cysteine residues are freely surface-accessible. However, a different conformation of the connecting loops, which would bring the remaining cysteine residues into spatial vicinity, can also be proposed (Figure 3). When applying two disulphide bonds as special restraints in the comparative modelling calculation, a structure with four intra-molecular disulphide bonds was inferred. Importantly, for *O. viverrini* c962, an N-terminal peptide of 94 amino acids, including two cysteine residues, was excluded from comparative modelling, due to the restrictions resulting from the template structure. Indeed, a limitation of the homology modelling approach for the *de novo*-determination of protein structures is the fact that, since homology models only extend to the boundaries of overlap between the template and the target protein in the alignment, the effects of N- or C-terminal peptides of the target on the fold are not considered. Experimental studies of the tertiary structures of different groups of SCP/TAPS will assist substantially in enhancing our knowledge of the structure-function relationships of these proteins.

### Developmental regulation of SCP/TAPS transcription

Levels of transcription of molecules encoding SCP/TAPS proteins were investigated in the juvenile and adult stages of both *O. viverrini* and *F. hepatica*, two key representatives of the Trematoda. In *O. viverrini*, significant (*p*<0.001) differences in transcription were recorded for eight distinct molecules encoding SCP/TAPS (seven single- and one double-domain SCP/TAPS) (Tables S1 and S2), of which four were up-regulated in the adult stage (Table S1). Conversely, in *F. hepatica*, transcripts encoding six distinct (i.e. four single- and two double-domain) SCP/TAPS were significantly up-regulated in the juvenile stage (*p*<0.001) (Tables S1 and S2), thus suggesting that SCP/TAPS proteins might play distinct roles during the infection of and/or the establishment in the mammalian hosts of trematode species with distinct biologies. For example, excysted juveniles of *O. viverrini* migrate from the duodenum through the ampulla of Vater and the common bile duct to the intra-hepatic bile ducts, where they develop into adult flukes [68], [69], whereas *F. hepatica* juveniles burrow through the intestinal wall and migrate through the peritoneal cavity and the liver capsule to then mature to adult flukes in the bile ducts [70]. Based on this knowledge, it is tempting to speculate that, in *F. hepatica*, the up-regulation of SCP/TAPS transcripts shown in the juvenile stages may favour the successful migration of the parasites through the host tissues, as hypothesized previously for the larval stages of the hookworms *A. caninum* and *N. americanus* [11], [14], [15], [71], whereas nothing is known about the molecular mechanisms that determine the up-regulation of such transcripts in the juvenile and adult stages of *O. viverrini*. Similar profiles of developmental regulation of genes encoding SCP/TAPS were observed previously in *S. mansoni* [21]. In this blood fluke, real-time PCR analysis of molecules encoding SmVALs revealed variable profiles, which included transcripts up-regulated in the developmental stage involved in the invasion of the intermediate (i.e. miracidium) or definitive (i.e. schistosomule) hosts and other transcripts ubiquitously expressed in all of the developmental stages studied (i.e. eggs, cercariae, miracidia, schistosomules, and adult males and females) [21]. In another study [72], an SCP/TAPS-encoding transcript was shown to be up-regulated in the vitelline tissues of *S. japonicum*, suggesting an involvement in reproductive pathways within the female worm. The developmental regulation of genes encoding SCP/TAPS throughout the life cycle of both *O. viverrini* and *S. mansoni* suggests that these molecules play diverse, but critical roles in the fundamental biology of these organisms. In the future, knowledge of the levels of transcription of genes encoding SCP/TAPS in other developmental stages of *O. viverrini*, as well as the localisation of SCP/TAPS proteins in the tissues of different developmental stages of parasitic trematodes, will assist in improving our understanding of the function of these molecules in these and other parasitic helminths.

### Genetic interactions of SCP/TAPS homologues

Networking predicted that 16 mouse homologues/orthologues of trematode SCP/TAPS genes/transcripts, including those encoding the glioma pathogenesis-related protein GLIPR-1, Golgi-associated pathogenesis protein GLIPR-2 and cysteine-rich secretory proteins CRISP-1 and CRISP-2, interact with a total number of 391 other genes (Table S3). While little is known about these interactions at this point, interestingly, previous studies [5], [73] have shown that the transcription of a *glipr-1* orthologue is high in human glioblastoma multiforme/astrocytoma and glioma cell lines, but not detectable in other neuronal cancer cell lines or in normal brain tissue. Although this link between up-regulated transcription and glioblastoma multiforme/astrocytoma remains to be proven, it is tempting to propose that some SCP/TAPS proteins from *O. viverrini* (a carcinogen; [74], [75]) are involved in the pathogenesis of cholangiocarcinoma in chronically infected humans. This hypothesis warrants testing. In the first instance, studies could explore, for instance, the morphological and molecular alterations in human bile duct cell lines exposed to various SCP/TAPS proteins derived from the parasite.

### Concluding remarks

Supported by the availability of the entire genome sequence of schistosomes [29], [30], recent advances in functional genomics provide unprecedented opportunities for fundamental investigations of SCP/TAPS proteins in different species and developmental stages of parasitic trematodes. Given that the life cycle of a range of parasitic trematodes (including *Schistosoma* spp.) can be maintained under laboratory conditions [76], [77], gene manipulation and/or silencing approaches, including transgenesis and RNA interference (RNAi) [78]–[80], could be employed for investigations of functional aspects of genes encoding SCP/TAPS proteins in parasitic trematodes. Such fundamental insights will enhance the understanding of key aspects of the biology of parasitic trematodes of socio-economic importance, such as pathways linked to the infection in the host and host-parasite interactions, and could provide the basis for important biotechnological outcomes, such as the development of novel strategies for the control of trematodiases.

## Supporting Information

Figure S1 **Structure-based alignment of full-length amino acid sequences of putative single-domain SCP/TAPS proteins predicted from the transcriptomes of** ***Clonorchis sinensis*** **,** ***Opisthorchis viverrini*** **,** ***Fasciola hepatica*** **and** ***Fasciola gigantica*** **(liver flukes), and the genomes of** ***Schistosoma mansoni*** **,** ***S. japonicum*** **and** ***S. haematobium*** **(blood flukes).**
(DOC)

Figure S2 **Structure-based alignment of full-length amino acid sequences of putative double-domain SCP/TAPS proteins predicted from the transcriptomes of** ***Clonorchis sinensis*** **,** ***Opisthorchis viverrini*** **,** ***Fasciola hepatica*** **and** ***Fasciola gigantica*** **(liver flukes), and the genomes of** ***Schistosoma mansoni*** **,** ***S. japonicum*** **and** ***S. haematobium*** **(blood flukes).**
(DOC)

Table S1 **A summary of the characteristics of putative single-domain SCP/TAPS predicted from the transcriptomic datasets from** ***Clonorchis sinensis*** **,** ***Opisthorchis viverrini*** **,** ***Fasciola hepatica*** **and** ***F. gigantica*** **(sequence data is available for download from** http://www.gasserlab.org/ **) and from the genomic datasets from** ***Schistosoma mansoni*** **,** ***S. japonicum*** **and** ***S. haematobium*** **.**
(DOC)

Table S2 **A summary of the characteristics of putative double(SCP-extracellular)-domain SCP/TAPS predicted from the transcriptomic datasets from** ***Clonorchis sinensis*** **,** ***Opisthorchis viverrini*** **,** ***Fasciola hepatica*** **and** ***F. gigantica*** **(sequence data is available for download from** http://www.gasserlab.org/ **) and from the genomic datasets from** ***Schistosoma mansoni*** **,** ***S. japonicum*** **and** ***S. haematobium*** **.**
(DOC)

Table S3 **A list of 16** ***Mus musculus*** **homologues/orthologues (left column) of trematode genes encoding SCP/TAPS proteins and corresponding interacting genes (right column), listed according to decreasing cut-off scores.**
(XLS)
