# Supplementary material for: Insights into SCP/TAPS Proteins of Liver Flukes Based on Large-Scale Bioinformatic Analyses of Sequence Datasets
Source: PLoS One. 2012 Feb 22;7(2):e31164. doi: 10.1371/journal.pone.0031164 (PMC3284463; doi:10.1371/journal.pone.0031164)
Supplement: Figure S1 — Structure-based alignment of full-length amino acid sequences of putative single-domain SCP/TAPS proteins predicted from the transcriptomes of Clonorchis sinensis , Opisthorchis viverrini , Fasciola hepatica and Fasciola gigantica (liver flukes), and the genomes of Schistosoma mansoni , S. japonicum and S. haematobium (blood flukes). (DOC) [file pone.0031164.s001.doc]

**Supplementary Figure 1.** Structure-based alignment of full-length amino acid sequences of putative single-domain SCP/TAPS proteins predicted from the transcriptomes of *Clonorchis sinensis*, *Opisthorchis viverrini*, *Fasciola hepatica* and *Fasciola gigantica* (liver flukes), and the genomes of *Schistosoma mansoni*, *S. japonicum* and *S. haematobium* (blood flukes).

**Group 1**

|---| CAP3 |--| CAP4

>S. japonicum GLIPR 1.4 ---------------MIQNSMLSL**C**FMIHLLYQPTLLVAL--DKDLTYLLNAHNTIRRYARN**C**NITGQPQARH---------------------LTDLKWSPELAMKALQLSRT**C**NFRYSQVKSSRFGV----VGQNIAAYAN

>S. mansoni Smp002070 ----------------MFKIVLVS**C**LLFLFTSLYVETKL---SEGQRAIYNFHKKVRKDVKN**C**RIPGQPPAKN---------------------LTKLKWNKLLANKAKQQAKR**C**KYDSNDPNDFIIGDF-ESIGQNLADYPT

>O. viverrini c962 TKISGSKSQQSGRSHNMLREKVAV**C**ILVFTSLILSSKELS--QKDKDTLLRMHNEARDKIRS**C**KLPGQPPVKGT--------------------YKPMVWDDEIAAYAQNWSQR**C**VLEHGGKPGNT--------GQNLAGVGS

>C. sinensis c4257 ----RFKTFNNPTHFDRTMSLLHL**C**LLSIVVLSSQVN**C**QTT-ATWVVAMLEQHNEARANVSR**C**IVPGLPPAKR---------------------LMPLMWDAALQRQAQLHANK**C**TLDEASMDDRKTTRFSH-VGQASSTGNV

>F. hepatica c6577 ---VLENSAWSFSMWHQMFLPIWL**C**VIIAEFQTVLAQEPNAADELNTRMLKEHNAIRQKALD**C**KIAGQPQAAK---------------------MPMLTYDQELADAARKWALE**C**RAERSSSADRVTKKWGLN-GQNVASSTD

>S. mansoni Smp193710 -----MSKGINMTASWTKTRYHRMVLTLLLGI**C**FLEAVFGMTAEEAQLILQVHNEHRAYRNL**C**GEKGIVPAEEI--------------------LQPLEWDDELAAAAQSWSKK**C**NPYEEEPIGNVGKWN--SVGRNSVIQSE

>S. haematobium cA06447 ---------MNNNHSLR**C**ISIMVILLFVIIIL**C**Q**C**KKLKL--PKEVREVFQLHKYYRNSIRF**C**QMPNQPPAKR---------------------MSKLKWNTYLAEKAQLSASR**C**DYSYDSPSDMNFDEFG-TVAQNIADSPT

>S. mansoni Smp123090 ----------------MNKNHPLHFVIFSLFV**C**TSNGAKKNLTQKLETIHRLHTYYRNSILL**C**KVPTQPPAED---------------------MEVLRWNDILANNAQQVANK**C**DLNFDLVNDKLLEHF-ESIGQNVAESDT

>S. haematobium cA06474 ----------------MIKLLIQSSLMYVLYIL**C**SDTQT---THDERTLYNKHVDYRRLLLK**C**KVPGQPKPIYP--------------------EEAFSWDNDLEDLANRQVVA**C**NLSTEYKKAEVEKEYRDTIGINTADNLN

>S. mansoni Smp001890 -----------MNNNRYTKYNYIIIIILFFIIIISH**C**KKLKLPKEVREVFQLHKYYRNSIRF**C**QMPKQPPAKY---------------------MSKLQWNKHLAEKAQLTASR**C**DYSYDSPSDMRFEEFS-SVAQNIADSPT

>C. sinensis c1247 KHQALKSQQSARSHNMLGEKIGVYTLILSILILPSEELS---QKDKDTLLRMHNEARDKIRS**C**KLPGQPPVKGP--------------------YLPMVWDDEIAEFAQSWSEQ**C**VLKHGGKPDHT--------GQNIAGVWG

>S. japonicum Pep.inhibitor1.3 ----------------MSKLLTLLFAIYFVDAVIKEHDV---GDLRNLLLALHNDERNVRSS**C**EYADIVPAEEK--------------------LDELTWDNELAADAQRFADQ**C**KKYVKQSVRSVGKWE--SVGQNAINVTE

>S. mansoni Smp127130 ----------------MTKILIQSSLMYLFYILYGNTQT---THEGRTLFNKHTDYRRLLLK**C**KVPEQPRPFYS--------------------EGELSWDDELEELAGRQASV**C**NLSSEYKKTEIEKEYR---GQIGINTAD

>S. mansoni Smp123550 ----------------MKFKQILYIFLLSIYIKVKIVFTQPKDAQLRKLLQYHNELRRNLTA**C**KLEGQPPAKN---------------------LLDLKWDNELASKAKDLANE**C**YFHHNDVDLPEKWQY---IGQNIAGYQT

>S. japonicum GLIPR 1.3 ----------------MKFIQLLILFVYTVFQTIINGKLT---PDLQKLLDLHNQVRRSLTA**C**TFDRQQPPAKH--------------------LPDLIWDEELAAKAKDLANE**C**YFHHNDVDLKHKWEY---VGQNIAGYQT

>S. mansoni Smp123540 ----------------MQQNLFFSFYSIIFLSEFISTIKLT--KDQDYLLKAHNRIRQYARS**C**NITGQPQAKR---------------------ILNLVWDDQLALKATELSKT**C**NFRFSNVTTYKFKD----VGQNIAGYAN

>S. japonicum GLIPR 1.2 ----------------------------------MIYGKQRLNAKSAELLRLHNKYRQDLVD**C**KVEGQPPAKY---------------------MSKLRWNHELAKQAKTLASK**C**ILKHKRASS-KRFEW---VGQNMAIYPS

>F. gigantica c566 ----------------------------IVSNFVVNGRPSENDALNKFMLQKHNELRSNLTK**C**NVRGQPPPVEM---------------------PMLRYDQELADLAQQWVDQ**C**KIGYDPDGIPTTSSWKNDVGQNWIGFYS

>F. gigantica c7108 -------------------------VIIAEFQTVLAQEPNAADELNTRMLKEHNAIRQKALD**C**KITGQPQAAK---------------------LPMLTYDQELADGARKWALA**C**KAQRSSLADRVTKKWGLNVGQNVASSTD

>O. viverrini c4326 ------------------------------------------TMLEQHNEARANVSR-----**C**IVPGLPPPKS---------------------LKPLIWDATLQRQAQLHASK**C**TLDEAPMNERTTTRFS-SVGQAATTGNV

>F. hepatica c4375 STQRHPTHGPNRIRLNMNSPLWF**C**FVSLILPHATVSGAPTSTDELNTLMLQLHNDARKKVLT**C**QLAGQPQAKS---------------------MPDLIYDQGIADKAQTWADT**C**TVGHDTYDARKTATFP-SVGQNFAGNSD

>S. haematobium cA08594 -----MIKGINMTASSTNTRYQRMVPTLLLAV**C**FVEAVFGLDAQEAQLMLKVHNEHREYRSK**C**GETGIVPAEEL--------------------LQPLEWDDELEAAAQRWSQK**C**DAYDDEPIGSVGKFDS-VGRNTFVISEL

>S. mansoni Smp160250 -----MTKGINMTASSTETRYHRMVLTLLLGI**C**FLEAVFGMTAEEVQLILQVHNEHRAYRNL**C**DKKDIVPAEEI--------------------LQPLEWDDELAAAAQRWSKK**C**NPFEEEPIGNVGKW--NSVGRNSVIQSE

>S. mansoni Smp159280 --------------MRSTIL**C**LT**C**TLFILSFFNENVY**C**NNALDKNSRELLELHRKYRQDLVD**C**KVDGQPPAKY--------------------MSPLVTYYSIISSNIKHVTLI**C**RSGIXXXXT**C**TKY---------------

>S. mansoni Smp159290 --------------MRSTIL**C**LT**C**TLFILSFFNENVY**C**NNALDKNSRELLELHRKYRQDLVD**C**KVDGQPPAKY---------------------MSPLKWNHDLARQAQSLANK**C**ILRHDKRHSNQFSW----VGQNIALHPT

>S. mansoni Smp002630 -------------------MFKKLIIYALINYVYGANTNEKLDKNSEELLELHRKYRQDLVD**C**KVDGQPPAKY---------------------MSPLKWNHDLARQAQSLAIN**C**TLQHDKRYSKQFIW----VGQNIALHPT

>S. mansoni Smp193680 --------------MFLREENVTLVVHILMILVNMIYANGGLDAKSEELLNLHRKYRQDLVD**C**KVDGQPPAKY---------------------MSPLKWNHDLARQAQSLANK**C**ILRHDKRHSNQFSW----VGQNIALHPT

>S. mansoni Smp078490 --------------MYSNLL**C**LMFTLFILLFFHEDVY**C**NKDLNKNSRKLLALHRKYRQDLVD**C**KVNGQPPAEY---------------------MSKLKWNYDLAQQAQSLASY**C**ILRQGKPRSKKFTW----VGQNIAFFST

>S. mansoni Smp139450 --------------MHSNLL**C**LT**C**TLFILLFFIENVY**C**KKDLNKNSRKLLVLHRRFRQDLVD**C**KVNGQPPAKY---------------------MSKLKWNYDLAQQAQSLANY**C**ILRQGKPHSKKFIL----VGQNMAFAST

>S. mansoni Smp141550 -----------MTASSTKTRYQRMMLTLLLGI**C**FVGAVFGLDPQEAQLMLRVHNEHRAYRKL**C**GEEDIVPAEEILQ--------------------PLEWDDKLAAAAQSWSEK**C**NPFDEEPIGNVGKWDSV--GRNSAIHSE

>S. mansoni Smp141560 -----------MTASSTETRYQRMVLTLLLGI**C**VVGAVFGLDPQEAQLMLRVHNEHRAYRKL**C**GEEDIVPAEEILQ--------------------PLKWDDELAAAAQSWSVK**C**NPFDEEPIGNVGKWDSV--GRNSAIQSE

>S. japonicum expressed protein ---------------MIKTILAS**C**LILLFTSQYVETR----LPLELEQIYKFHKKVRKGLLN**C**KIPGQPPARFLE---------------------KLKWSKLLARKAQKQANK**C**NFNSTDPDDFV-----------------

>F. hepatica c8831 --SALYISANNGNMKKGILLVITLYELLIVSNFVANGRPSENDALNKFMLQKHNELRSNLTK**C**NVRGQPPPVEMP---------------------MLRYDQELADLAQQWVDQ**C**KIGYDPDGIPT-----------------

>S. haematobium cA08595 ------------------TRYQRMVPTLLLAV**C**FVEAVFGLDAQEAQLMLKVHNEHREYRSK**C**GETGIVPAEELLQ--------------------PLEWDDELEAAAQRWSQK**C**DAYDDEPIGSVGKFDSV--GRNTFVISE

>C. sinensis c1246 ----NEKSFIASYQMIRSKTTTRFLWFLLSYGLLSAPSIALSGDWQSEMVRLHNEARDKILT**C**SVSGQPPAKSMPH---------------------LSWHAGLAEKAQQLADQ**C**RVGHDKAEERKVPDFDYV-GQNWAGVQD

>O. viverrini c2686 ---------------QMIQPTTRFLWSLLL**C**GLLSAP**C**IALSGDWQSEMVRLHNEARDKILS**C**SVPGQPPAKSMPH---------------------LVWHDGLAQKAQQLADQ**C**RVGHDTAEERKVPDFDYV-GQNWAGAQD

>S. haematobium cA08596 ---------------------------------------GLDAQEAQLMLKVHNEHREYRSK**C**GETGIVPAEELLQ--------------------PLEWDDELEAAAQRWSQK**C**DAYDDEPIGSVGKFDSV--GRNTFVISE

>S. japonicum Pep.inhibitor1.1 IKNSSELNKQLREMGSQMIISVIIVFSIVVKASTKINGEKRLNAKSAELLRLHNKYRQDLVD**C**KVDGQPPAKY---------------------LPQLRWNHELAKQAKTLASK**C**ILRHEMASSRRFDW----VGQNMAIYPS

>C. sinensis c2194 --------------KNSMFNVRFSGYLLLLVGFIQPSREQTTEDQRQKFLKFHNDLRDKIRK**C**ELPGQPPAKA--------------------PYEPMVWDTAVEAQAQKWADK**C**LFSHGETD---------GVGQNIAIAGS

>C. sinensis c353 -----------------EPMTMRTN**C**IFFYVTTVFS**C**FVFSVSYDPKDFVKLH-DQGRVRLQKGEVLGQP**C**ARNMP--------------------PVVWDNELAEKAQKWASK**C**QAGHDSNSERKTKKFDLV-GQNWAGGYD

>O. viverrini c1174 -----RFLAIRVPDI**C**PHATRMHINFILFYVTAVSY**C**IVFSKTPKLTQFVDLHND-GRLRVQKGEIPGHP**C**AKYMP--------------------LVKWDKGLARKAQKWANK**C**KPEHDNRKNRKTSKFSVV-GQNWAIGYD

>O. viverrini c15215 ---------------------------------------------------LHND-GRLRVQKGEVPGHP**C**ARYMPPVVGLIIS**C**YRYENYSPNQ---EWDEELARKAQEWANK**C**KAEHNX----------------------

>S. haematobium cA01429 -------------------MFKISLISYLLLLFTFLYVETKLSGELREIYRFHKKVRKDVKN**C**KIPGQPPAVK---------------------LAKMKWNKLLADKAKQQVKR**C**QYDSNDPNDFIIGDFESI-GQNLGDYPT

>O. viverrini c6515 Q**C**IYIHIDTSEAHFAINMFKVRFAGYVLLLVGFTQPSREQTTDAQRQKLLKYHNDLRDKIRS**C**QLPGQPPVKGPYE--------------------PMVWDSDVEAQAQRWADN**C**KFAH---------------GELEGVGQN

>F. hepatica c8830 -----------------------------------------------------------------------------------------------------------------------------------------------

>S. mansoni Smp116210 -----------------------------------------------------------------------------------------------------------------------------------------------

>S. mansoni Smp118160 -----------------------------------------------------------------------------------------------------------------------------------------------

>S. mansoni Smp120240 -----------------------------------------------------------------------------------------------------------------------------------------------

>S. mansoni Smp100560 -----------------------------------------------------------------------------------------------------------------------------------------------

**Group 2**

>C. sinensis c3048 FRNRQWNHSQGLLLKMSSWNKVAQVLLGWVIFIQL**C**ALTQAIMYDDEFFLEEHNNYRRMLLDGEVPNQPIPKH---------------------LPPLQWSSHLKDSARRWTER**C**IYEPENNPEQG-----------------

>S. mansoni Smp002060 ----------------MMIKLSLIFLLNILIINADK------SSTKELIFNFHNKIREDVFKGVLSGQPKAEK---------------------DVQTEWNKLLAKLAKGHVQK**C**ILDSGDLGKLYVGKFD-SVGQTVAEHTS

>S. japonicum GLIPR 1.5 --------------------------------------MFTYEGTKVRLLTLHNDARKSVVEGKLMGQPMAIS---------------------MEPLKWDKELERKAQILADN**C**SFAHDNVTNRSTSSFEH-VGQNIARADS

>S. japonicum AllergenV5Tpx1.2 ------------------------------------------------------------------------------------------------MKSWDSELAELASRQVAA**C**NLSSRYNKEEVERIYRDTVGINTADDAD

>S. haematobium cA08597 ---------IYMTASLTKTRYQRMVPTLLLAI**C**SMEAVFGLNAEEEQLMLQVHNEHRRYRSKYNETDIMPAEAI--------------------LQPLEWNDELEAAAQRWSQK**C**DAYDEKPIGS--------VGKFDSVGRN

>S. mansoni Smp176180 ---------------MSFKFHVSTIIFLT**C**FLL**C**S**C**VHAKMNDTIREQLLTMHNVVRELAKFGLIPRQPEAVH---------------------MKLLKWNMELERKAQNLSDQ**C**KSEHDEIEQRRIPEFQN-VGQTWVGTYT

>S. haematobium cC00745 --------------------------------------------MRDEFIAMHNVVRQAVKYGLIPGQPGAVH---------------------MGLLKWNTGLEMKAQNFSDQ**C**KLGHDKESERKIGNFTY-VGQNRALTPT

>S. mansoni Smp120670 --------------------------------------------MRDYFIAMHNVVRQAVKYGLIPGQPEARH---------------------MNLLKWNTELAKKAQNFSDQ**C**QLGHDKDEERKIPGFEY-VGQNWALTPR

>S. haematobium cA08598 HIHSST**C**RKSSHLEMTAK**C**QSSLTALTLV**C**LFL**CCC**VHAAIDKATREKLLTLHNNARTSVLHGWLEGQPMARS---------------------IKPLKWNVELEEKAQMLADT**C**YFGPDNAIERKVPNFT-NVGQNWAGANT

>S. mansoni Smp070250 LIYSSSYWKAIHFEMTVK**C**QSSLTALTTL**C**LLV**CC**GVHAAMDNATREKLLKLHNNARISVMHGQLEGQPIATS---------------------IKPLKWNMELEKKAQMLADT**C**YFGPDSAIERKVPGFT-NVGQNWAGAST

>S. mansoni Smp179480 LIYSSS**C**WKAIHLEMTVK**C**QSSLTALTTL**C**LLV**CCC**VHAAMDNATREKLLKLHNNARISVMHGRLEGQPIAKS---------------------IKPLKWNMELEKKAQMLADT**C**YFGPDSAIERKVPGFT-NVGQNWAGAST

>S. japonicum GLIPR 1.1 --------------MHQKSYYSPVVVLLVGILVSSRVDGLMDDKMREQLLTLHNTVRAALRNGRLTGQPRAIS---------------------IKPLKWNMELESKAQSLSDQ**C**RVGHDTYDARKTPEFS-LVGQNWAGSKD

>S. japonicum Allergen V5Tpx1.3 ----------------------------------------MDNRMRTQLLSLHNTARAAVRNGQLSGQPIAVS---------------------MKLLKWNTELEMKAQFLSDQ**C**VFGHDTNNDRKTSQFP-YVGQNWAGSQD

>S. haematobium cA09053 ---------------MSSNSEFSSVVLLV**C**SLV**CCC**VHAQLDDAMRNELLALHNKARQSVQNGQLVGQPIAVS---------------------IKPLKWNVELETKAQILSDQ**C**RVGHDTNADRKIPAFQ-YVGQNWAGAKD

>S. mansoni Smp154260 ---------------MSSNSDFSSVVLLL**C**LLV**CCC**VHAKLDDAMRNELLTLHNEARQAVRNGQLFGQPIAVS---------------------IKPLKWNVELERKAQILSDQ**C**RVGHDTNADRQIPEFQ-YVGQNWAGATD

>S. mansoni Smp176160 ---------------MSSNSNFSSVVLLL**C**LLV**CCC**VHAKLDDAMRNELLTLHNEARQAVRNGQLFGQPIAVS---------------------IKPLKWNVELERKAQILSDQ**C**RVGHDTNADRQIPEFQ-YVGQNWAGATD

>S. mansoni Smp154290 ---------------MSSYSDFSSVVLLVYLLV**CCC**VHAKLDDTMRNELLTLHNKARQSVRNGQLFGQPRAVS---------------------IKPLQWNVELERKAQNLSDR**C**QVGHDTNADRKIPKFQ-YVGQNWAGAKD

>S. mansoni Smp070240 ----------------MFYTIQVNFIIFLVLGYIYENLAYRWYTQNSELLALHNAYRRNIKYGNVRDQPQAMSML---------------------KLTWSHKLAEMAQEWALQ**C**VPRRSNMTMRKGSKWTYV-GQSIAFVPK

>S. japonicum Pep. Inhibitor1.2 ------------------MHIVQFSVVLLVLVNIYKNSAVNWHIEDEEILALHNAYREAVKFGRVRDQPKAISMS---------------------KLQWSYQLAKLAENWTIH**C**IPKTSGLKFRNSSKWTYV-GQNVAVVSK

>O. viverrini c7991 ----------RITFKSTRFGMSVKRIL**C**FV**C**LVVLQQISAMFWRDAKLFVDEHNRYRKMLVDGELTNQPTARYM---------------------RILSWDRLLSRNAQRLASE**C**RVGHDSGSERATPTFPLV-GQNWAGTDN

>O. viverrini c17638 -----------------------------------------------------------------------------------------------------------------------HNSNENRRTSKFGRVGQNLAMGYD

>O. viverrini c18160 ----------------------------------------------------------------------------------------------------------------------------------------NVAIASS

>O. viverrini c15680 ------------------------------------------------------------------------------------------------------------------------------------IYGENLAFSYG

>C. sinensis c14383 ----------VAFTSTRFGMRVKRIL**C**FV**C**LAVLLQTSAM**C**-WRDAKLFVDEHNRYRKMLLDGEVANQPTARYMTRYQRMVPTLLLAV**C**FVEAVFHMLSWDRLLSRNAQRLASE**C**RVGHDSGSERATPTFPL-VGQNWAGTDN

>S. haematobium cA08206 -------MQAVHTNHLFNMHKTQFSLIVLVLLNVYGNRAIFWNIENSELLVLHNAYRRDIKYGTVLDQPKADSMLKLQ---------WSENIAITRGGFHNHKLAKLAQAWAFH**C**VPTRSNLTMRDGSKWTY-VGQNIAVVSK

>F. gigantica c25411 -------------------------------LTLYHISMSTTRVERRRFVDEHNKYRTMLMAGKVSGQPRASSMK---------------------MLTWDEELARKAQFLANK**C**QVGHDTLTERQTKEFHWV-GQNWAGTFN

>C. sinensis c4741 TQGAVTPQEYSSNNVQEQPSEPVYTITDRATGEVINVKVHPEMLRDSEFLDEHNFYRRRLRDGTDPDGTHVPGLA---------------------DLIWDEALAEE**C**EKWAKT**C**VYQYAQNIA**C**---------EENLASTVK

>O. viverrini c4477 LRNRLLSRMQPSSNGVQNAEKPVYTIVDQNTGENITVDVSPEVPKDSEFLDEHNFYRQRLREGTDPEGYHVPGLA---------------------DLIWDRELAAE**C**RRWAET**C**VYQYAQNINA---------EENLASTTN

>C. sinensis c7351 RPQNEQTGQQNQLQVPPEKQENKLSFTNPLTGKKYTVRALERVFSARQFLDRHNFYRGRVRNGQEAGRYAVPGLR---------------------NLLWEAKLAEGARAWAER**C**IYKHPDSLDV---------GENIGYSSG

>C. sinensis c5350 --------SYRGVEESENPSLGTDTFFDPEHKAYETVHYYARFYTAKQFLDKHNFYRRRLLHGTEPDGFRAKKMP---------------------DLVWDETLYEEAKAHAQK**C**IFAHDPEDKVY--------GENLALSYG

>C. sinensis c13355 ----------------------------------------------------------------------------------------------------------------ES**C**VQKHVLPTGQ---------GENLAVTTA

>O. viverrini c5997 ---------------------------------------------------------------------------------------------------------AAAKNVAES**C**AQKHVIPVGQ---------GENLAVTTT

>F. hepatica c6081 -**CCC**IDYEAMGKKDKVTRPRPKPDTRTTQSSSDTEDFSGPRVYNTREEYLKGHNYYRRLIRDGRKTSQPRSAN---------------------LYDMVWNNTLERKAYEMSLR**C**TFNHEET------------GENIYAGTS

>S. japonicum PRP4 -------------MHPISKSSIKALLSI**C**LLV**C**TYVHAWKDN-KSPSRLLALHNNVRKSVVNGELAGQPMAVS---------------------LKSLEWDKELERKAQILADNGSFTHDNVTNRSTSSFAY-VGQNIAGANN

>O. viverrini c3648 -----------------------------------------------------------------------------------------------------------------------------------------------

>C. sinensis c10021 -----------------------------------------------------------------------------------------------------------------------------------------------

>S. haematobium cA06111 --------------------------------------- MNDTIRGQLLTMHNVVRQLAMYGLIPGQPKAVYMN-------------------PLLVWENT-----------------------------------------

>O. viverrini c18119 -----------------------------------------------------------------------------------------------------------------------------------------------

>S. mansoni Smp176170 ---------------MSSNSNFSSVVLLL**C**LLA**CCC**VHAKLDDAMRNELLTLHNEARQAVRNGQLFGQPIAVS---------------------IKPLTSSTFLNMDFAVFSSNSTHMNIGIDHN------------------

>O. viverrini c19644 ------------RAILSM**C**EIKLTAYVLLLT**C**SIQLSRAKTTQEQKTKFLDMHNELREKIRK**C**TLSGQPPVRGN--------------------YELMTWDEAVEAQAQX---------------------------------

**Group 3**

>O. viverrini c6176 ------------------------------------ISEAMDEQFNRE**C**LEEHNRLRALHG-**C**P--------------------------------PLELDVELARNAQAHSEKMANENVMHH**C**LSRGH-----GENL**C**IREG

>C. sinensis c2042 RWEAMVINYLLHNPASLTAFQRIKFTK**C**PNLTRSSLISEAMDEQFNRE**C**LEEHNRLRALHG-**C**P--------------------------------PLELDVELAQNAQTHSEKMASKNVMHH**C**LSQGH-----GENL**C**IREG

>S. haematobium cA01987 ------------------------------------MPSDLDIEFNEE**C**IIEHNRLRALHG-**C**P--------------------------------ELILDYELAKDAQKYAEHLASVNELEH**C**TDTDS-----GENLAFFTT

>S. mansoni Smp163400 ------------------------------------MPNDFDIEFNEE**C**INEHNRLRALHG-**C**P--------------------------------ALILNYELAKDAQKYAEHLASVNELEH**C**TDTDS-----GENLAFFTT

>S. japonicum GAPR 1.3 ------------------------------------MS**C**NIDIEFNEE**C**ILEHNRLRALHG-**C**P--------------------------------ELTLDYELASAAQQYAEYLASVNELEH**C**TDTDS-----GENLAFFTT

>S. mansoni Smp124060 ---------------------------------------MVDEQLNHDALNEHNRLRALHG-**C**P--------------------------------PLKYDRRLAREAQAWAENLARLKIMKHSI**C**DEY-----GENLASAQS

>S. japonicum GAPR 1.1 ---------------------------------------MIDKQLNREALDEHNRLRALHG-**C**P--------------------------------PLQYDERLARDAQSWAENLARLKILKHSI**C**DEY-----GENLATSMS

>F. hepatica c9426 --------------------------------------------LNAEFIDAHNEYRALHG-**C**G--------------------------------KLKFDMALARSAQKYAEQLAQLGYMNHSS**C**DGY-----GENLAARSS

>S. haematobium cA00481 ---------------------------------------MVDEQLNHDALNEHNRLRALHG-**C**P--------------------------------PLKYDSRLAREAQAWADNLARMKIMKHSI**C**DEY-----GENLATSQS

>F. gigantica c1042 ------------------------------------------------------------------------------------------------------------QKYAEQLAQLGYMNHSS**C**DGY-----GENLAARSS

>S. mansoni Smp124070 ---------------------------------------MIINKLNKEAIQAHNELRALHG-**C**P--------------------------------EISYDSKLASDSQKWAEHLASIN**C**LQHSKGDDY-----GENLAFQMS

>S. haematobium cA07983 ---------------------------------MNAYHFLHKFSYHTSVKY**C**KSTLRALHG-**C**P--------------------------------KISYDSKLASDSQKWAEHLASTN**C**MQHSKADGY-----GENLAFQMS

>C. sinensis c3765 FSHHISESQTTAKQPAVYRAFRFLADQLPFHWYSNKLENVAY**C**QNF**C**NGNYRIKNRRN**C**FTQQ---------------------------------ATRAARMRAFIVRFFIGSLSTTK**C**MRHSDMATY-----GENLAYR**C**I

>F. hepatica c10643 ---STGTRFRLFGVLYQPRSTPSAPNSSANSSILLNSRGRAMDSLNQEALTAHNRYRAMHG-**C**P--------------------------------PLAFDSALARSAQKWAEDLAGTQ**C**MRHSDQTTY-----GENLAFMGI

>O. viverrini c8913 ---------------------------------------------------NKLRERHG---**C**G--------------------------------PLSYDSSLARSAQLWAEELATTK**C**MRHSDMATY-----GENLAYR**C**I

>F. hepatica c1472 ANITLTKTNNNDTNLEMELEQNTPVSSPENVITAVKPTSEINEQFNQE**C**LEEHNRLRALHG-**C**P--------------------------------PLELDRNLAAKAQEHTEVMALKNQMHHRLSPNH-----GENLSMREG

>F. gigantica c3 ----------------------NTPISSPENVITVKPTSEINEQFNQE**C**LEEHNRLRALHG-**C**P--------------------------------PLELDRNLAAKAQEHTEVMALKNQMHHRLSPNH-----GENLSMREG

>F. gigantica c4035 VNFPLETQEQTFDVF**C**VSHNLFRGYEMGSAAGGLTLVSVSVDEPFNLE**C**LAEHNRLRKLHG-**C**P--------------------------------PILLDENLAKMAQLHAHRMAETQTFLSSGQKNI-----GENLWMH--

>S. haematobium cA03186 ---------------------------------------MINERFNQQAIREHNRLRSLHG-**C**G--------------------------------ELQLDEELMISAQKWAENLADAEKLYHSNYNDY-----GENLAFKMS

>S. mansoni Smp124050.1 ---------------------------------------MINERFNDQAIREHNRLRSLHG-**C**P--------------------------------ELQLDEDLMISAQKWAENLAAAEKLYHSNYNDY-----GENLAFKMS

>S. mansoni Smp124050.2 ---------------------------------------MINERFNDQAIREHNRLRSLHG-CP--------------------------------ELQLDEDLMISAQKWAENLAAAEKLYHSNYNDY-----GENLAFKMS

>S. mansoni Smp124050.3 ---------------------------------------MINERFNDQAIREHNRLRSLHG-CP--------------------------------ELQLDEDLMISAQKWAENLAAAEKLYHSNYNDY-----GENLAFKMS

>S. mansoni Smp124050.4 ---------------------------------------MINERFNDQAIREHNRLRSLHG-CP--------------------------------ELQLDEDLMISAQKWAENLAAAEKLYHSNYNDY-----GENLAFKMS

>S. japonicum GAPR 1.2 ---------------------------------------MINERFNQQAIREHNRLRSLHG-**C**P--------------------------------ELQLDEELMISAQKWAENLADAEKLYHSNHNGY-----GENLAFKMS

>C. sinensis c3166 ---------WHHTTPFRKLPYHGTIKGH**C**NTLLLDNTIDRMDRQLNDEAIAAHNQLRAAHG-**C**P--------------------------------KLTLDPELARGAQKWAEELAKTKRLQHSNDKRF-----GENLAYQQS

>O. viverrini c4390 --------------------------------LLDNIINRMDRQLNDEAITAHNQLRAAHG-**C**P--------------------------------KLTLDPELARGAQKWAEELARTKRLQHSNDKRF-----GENLAYQQS

>O. viverrini c18440 -------------------------------------QNGVQDAVFEDAINAHNEYRKVHG-**C**P--------------------------------ALTLDNKLAEGAQKYAERLAQLRKLIHSDSKEY-----GENLATSIS

>F. gigantica c3823 INPWTMTLSNRTLNNTLHLRLRFLLPPFWSISELPVSSNMVDRTFNNEAITAHNYFRSAHG-**C**P--------------------------------NIVYDAELAKQAQSWASHLAKNGNMQHSKAQGY-----GENLAYKWS

>O. viverrini c11939 -------------------------------------------------IEEHNRLRALHG-**C**A--------------------------------PLTLDPELARKAQLHAEELAAGKEHIQS--NEY-----GENVAMRKT

>F. hepatica c9115 -------------------------EMGSAAGGLTLVSVSVDEPFNLE**C**LAENNRLRKLHG-**C**P--------------------------------PILLDENLAKMAQLHAHRMAETQTFLSSGQKNI-----GENLWNNSG

>S. haematobium cA00727 ---------------------MISDDHRYDNQKIIKSNKIELDHFNEI**C**LKENNRLRQLHN-**C**P--------------------------------KLKLNYRLIKSAQIHSEYLQKLHQL----------------------

>C. sinensis c1770 LPNRH**C**IPRKTNVYPPVLGRVVFSHQPTNNQIVGLLFHVIMDLRFNEI**C**VQEQNRLRKLHG-**C**P--------------------------------PVRVDPQLVQKAQALANRMARSQTIMTFGDPTI-----GENFYLHVG

>O. viverrini c3766 -----------------K**C**PPT**C**SGTSSFPHIVGLIFHVIMDLRFNEL**C**VQEQNRLRKLHG-**C**P--------------------------------PVRVDPLLVQKAQTLANRMALSQTITTFGDPTV-----GENFYLNIG

>O. viverrini c11437 QTDTSAETYDVETWYSSLTMDKIIPVKRTKLSHPLNTETAHLEAFR**C**EVLHTHNRYRAMHG-VP--------------------------------DLKRSATLDALALNWAKELQMTGTPAYWEHEYGNSLV-GENVADRIT

>F. gigantica c4654 XDGRVKRVTSESFAEASDTITTESSTGPEAGGLVTGNSKKDLESFGEAVTQVHNTHRARHG-AP--------------------------------ALKYDPQLSELAQKWAEELVQMPRLSNSGYTFEGVRL-GENVLSRWS

>F. gigantica c12544 -------------------------LVYFGEDFNLNLTMAGMDDFIEE**C**LREHNAKRSLHN-AP--------------------------------ALRHSRALDKTAQDWAEQLISEEQIKNSSLSGRGEV--GESISMRTS

>S. haematobium cB00359 --------------------------------------MSLLDDFRED**C**FIAHNEKRLLHGV**C-**--------------------------------ALRHSLALDKTAQDWAEALLLEDGIKNSPLSSR--------------

>C. sinensis c8455 YAHV**C**PNIPRAVLSLITPQTTVYDNVGLFSTGYLPFAYTMLDTELNTQAIALHNQFREKHG-SP--------------------------------PLVYDAKLAQTAQNWAEQLAQTK**C**MRHSDMETY-----GENLAYKGA

>O. viverrini c2349 ----ILNIPRAVLSLITPQTTVYDKVGLFSTGYLPFAYTMLDTELNTQAIALHNQFREKHG-SP--------------------------------PLAYDAKLAQTAQNWAEQLAQTK**C**MRHSDMDTY-----GENLAYKGA

>C. sinensis c14468 -----------------------------------------------------------------------------------------------------------------------------------------------

>C. sinensis c13576 -----------------------------------------------------------------------------------------------------------------------------------------------

>F. gigantica c12861 -----------------------------------------------------------------------------------------------------------------------------------------------

>C. sinensis c6993 -----------------------------------------------------------------------------------------------------------------------------------------------

**Group 4**

>S. haematobium cA03835 ----------------------------------------MTKEQFNLHGIHHKLKDDNHDFRIFEQP--------------------------LLKHKRDNTLQQYVERYVKQ**C**NPSITLTKQYFNNTL--SV--NISDNKD

>S. haematobium cA06788 ----------------------------------------------------------------------------------------------MYSLDRNSIRSSHRRSFRESLGLSER**C**K**C**YWDHTPYL------------

>S. mansoni Smp035980 --------------------MILLLLISLILVSPGLGGQDEMAKFTSTLLQHHNWVRQTRNK**C**DSRWSTSEKKLEDF---------------------IWDDGLYVTAQQYAEK**C**ENIPSGLHDRTT**C**RWK-SVGQNVAQVSS

>S. mansoni Smp131370 ------------------------------------------------------------------------------------------------MLKRDNTLQQYVERYVKQ**C**DPSITLTKQYLNNTP--SA--NISDNRD

>S. haematobium cA07187 -------------------------------------------------------------------------------------------------------MAQQWADHLLQQSHLSNSGYVYRGMKV----GENLGSRWS
>O. viverrini c19841 --------------------------------------------------------------------------------------------------TWDEAVEAQAQKWSDK**C**IFGHGESEG-------------------

>S. japonicum Allergen V5Tpx1.1 -----------------------------------------------------------------------------------------------------------------------------------------------

>S. haematobium cA00413 -------------------------------------MINTEKSSKEMIFKFHNKVRKDVLKGVLSDQPKARKM---------------------PKLKWNELLAEKAKDHVKK**C**ILDSGNL------------GDLYVGKFD

>C. sinensis c2194 ----------------------------------------------------------------------------------------------------SVWRKLFIEKFVHNKLFGLPAFTX-------------------

**Group 1**

>S. japonicum GLIPR 1.4 -------------------------------------------------------------------------------AEIAMNQWIDEYKYYDFEKNI**C**--NEP**C**------GNYVQIVWQET------------------

>S. mansoni Smp002070 -------------------------------------------------------------------------------IEGAMKDWLEEYKNYNFEKNQ**C**--NGD**C**------KNYKQMVWNTT------------------

>O. viverrini c962 -------------------------------------------------------------------------------IDGGPYAWFGEHVNYNHRDHS**C**ASGRV**C**------GHYTQMVWQDS------------------

>C. sinensis c4257 -------------------------------------------------------------------------------AKNVLSRWFNEYKKLDIITNH**C**-NS**C**P**C**------RDFRMVISDQV------------------

>F. hepatica c6577 -------------------------------------------------------------------------------FQGVVDLWFIGHRHYDYANNQ**C**DRLYN**C**------NSYLQIVSAKT------------------

>S. mansoni Smp193710 -------------------------------------------------------------------------------LAGAVAYWMEESNIYKHNTDS**C**SSTFE**C**------NSYKQIVQAET------------------

>S. haematobium cA06447 -------------------------------------------------------------------------------IEKAVASWFVEYKSYSFNDNK**C**--NDT**C**------MQYKQIVKGEE------------------

>S. mansoni Smp123090 -------------------------------------------------------------------------------IKNAMENWFREYHNYNYETDK**C**--NGS**C**------SNYRQMVWAKT------------------

>S. haematobium cA06474 -------------------------------------------------------------------------------VLNAMETWFNEYQLYDYTKNK**C**KVPKD**C**------LHYKRIVWGSA------------------

>S. mansoni Smp001890 -------------------------------------------------------------------------------IEKAVASWFIEYKNYSFDDNT**C**--KDT**C**------MQYKQMVKGEE------------------

>C. sinensis c1247 -------------------------------------------------------------------------------IDGGPYAWFNEHVNYNHRNHS**C**APNRA**C**------GHYTQMVWQES------------------

>S. japonicum Pep.inhibitor1.3 -------------------------------------------------------------------------------IADAVRLWTKEANYYNHSLDT**C**MAGHD**C**------DSYKQMVQAET------------------

>S. mansoni Smp127130 ----------------------------------------------------------------------------NSDVINAVESWFNEYQLYDFTNNK**C**EVPED**C**------LHYKRIVWDKA------------------

>S. mansoni Smp123550 -------------------------------------------------------------------------------IEQAFDAWKDEYKQYNYYSKS**C**--SGV**C**------GHYTQLVWQNT------------------

>S. japonicum GLIPR 1.3 -------------------------------------------------------------------------------IDLAFDSWLSEYKMYHFYSTS**C**--SGV**C**------GHYTQLVWQNT------------------

>S. mansoni Smp123540 -------------------------------------------------------------------------------VQTAMDEWVNEYQYYDFDSNT**C**-NSKS**C**------GNYLQIVWQKT------------------

>S. japonicum GLIPR 1.2 -------------------------------------------------------------------------------IQEGVDAWFNEHKMFNYYGGK**C**---RQ**C**------LHYTQMVWANT------------------

>F. gigantica c566 -------------------------------------------------------------------------------FQKAFDLWFEGHLDYDYNRNS**C**--DTV**C**------HHYTQMVWAST------------------

>F. gigantica c7108 -------------------------------------------------------------------------------FKGVVDLWFIGHRHYDYVNNQ**C**DRVYN**C**------KGYLQIVSAKT------------------

>O. viverrini c4326 -------------------------------------------------------------------------------AKNVFARWFSEYKRLDFNTNY**C**NR**C**P-**C**------RDFRMALSDRV------------------

>F. hepatica c4375 -------------------------------------------------------------------------------FRSAFDSWFSENTNYMFDTNT**C**ASSKS**C**------GHYTQIVWAKT------------------

>S. haematobium cA08594 --------------------------------------------------------------------------------AEAVTYWMEESDHYDHKSDQ**C**EIPHQ**C**------DSYKQIVQAET------------------

>S. mansoni Smp160250 -------------------------------------------------------------------------------LAEAVAYWMEESNIYKHNTDF**C**SSTFG**C**------NSYKQIVQAET------------------

>S. mansoni Smp159280 -------------------------------------------------------------------------------NKIVYKLYKHILQLIDRSHYFLVHIP-----------FQMAWAKT------------------

>S. mansoni Smp159290 -------------------------------------------------------------------------------INSGVDAWFNEHKLYNYNMNN**C**P---Q**C**------LHYTQMAWAKT------------------

>S. mansoni Smp002630 -------------------------------------------------------------------------------IKSGVDAWFNEHKLYNYNTNN**C**P---Q**C**------LHYTQMAWAKT------------------

>S. mansoni Smp193680 -------------------------------------------------------------------------------IKSGVDAWFNEHKLYNYNTNN**C**P---Q**C**------LHYTQMAWAKT------------------

>S. mansoni Smp078490 -------------------------------------------------------------------------------INSAVDAWFNEHKLYNYSVNN**C**P---K**C**------VHYKQMVWAKT------------------

>S. mansoni Smp139450 -------------------------------------------------------------------------------IKSAVDAWFNEHKLYNFSVNN**C**P---Q**C**------LHYTQMVWAKT------------------

>S. mansoni Smp141550 -------------------------------------------------------------------------------LAEAVAYWMKESNYYDHNSDL**C**EPSHH**C**------NTYKQIVEAQT------------------

>S. mansoni Smp141560 -------------------------------------------------------------------------------LAEAVAYWMKESNYYDHNSDL**C**EPSHH**C**------NTYKQIVQAET------------------

>S. japonicum expressed protein ----------------------------------------------------------------------------------------------------------------------------------------------

>F. hepatica c8831 ----------------------------------------------------------------------------------------------------------------------------------------------

>S. haematobium cA08595 -------------------------------------------------------------------------------LAEAVTYWMEESDHYDHKSDQ**C**EIPHQ**C**------DSYKQIVQAET------------------

>C. sinensis c1246 -------------------------------------------------------------------------------IETAVRMWFEEHVNYDFSTGN**C**RQGM-**C**------GHYTQVVWAST------------------

>O. viverrini c2686 -------------------------------------------------------------------------------IETAVRMWFEEHVNYDFSTGN**C**RQGM-**C**------GHYTQVVWAST------------------

>S. haematobium cA08596 -------------------------------------------------------------------------------LAEAVTYWMEESDHYDHKSDQ**C**EIPHQ**C**------DSYKQIVQAET------------------

>S. japonicum Pep.inhibitor1.1 -------------------------------------------------------------------------------IQEGVDAWFNEHKMFNYYGGK**C**R---Q**C**------LHYIQMVWANT------------------

>C. sinensis c2194 -------------------------------------------------------------------------------VEVAVKLWADEYVNYDPASGG**C**KPSGE**C**------LHYTQMAWAAS------------------

>C. sinensis c353 -------------------------------------------------------------------------------LQGAFNAWFDEYRNYNYANRS**C**T-GV-**C**------GHYTQIVWNET------------------

>O. viverrini c1174 -------------------------------------------------------------------------------LKTAYDEWFVEYQDYNYTTRE**C**K-GM-**C**------GHYTQAVWNET------------------

>O. viverrini c15215 ----------------------------------------------------------------------------------------------------------------------------------------------

>S. haematobium cA01429 -----------------------------------------------------------------------------------TEGDSEERPYEAKSESE**C**NKSEN------------------------------------

>O. viverrini c6515 -------------------------------------------------------------------------AAVASNLELAVKLWVDEYMNYNYSSGS**C**KPMTE**C**------LHYTQMAWATS------------------

>F. hepatica c8830 ---------------------------------------------------------------------------------GPWLSLIEGHLDYDYKRNS**C**--DTV**C**------HHYTQMVWAST------------------

>S. mansoni Smp116210 ----------------------------------------------------------------------------------------------------------------------MAWAKT------------------

>S. mansoni Smp118160 ----------------------------------------------------------------------------------------------------------------------MAWAKT------------------

>S. mansoni Smp120240 ----------------------------------------------------------------------------------------------------------------------MAWAKT------------------

>S. mansoni Smp100560 ----------------------------------------------------------------------------------------------------------------------MAWAKT------------------

**Group 2**

>C. sinensis c3048 ----------------------------------------------------------------------------------PMELWFSEHWLYKMGQMKENETHLV------SEYVQMVNAKT------------------

>S. mansoni Smp002060 -------------------------------------------------------------------------------IQNILDTWLEEKNDYDLDKNT**C**E--NE**C**------GNYKQLVWANT------------------

>S. japonicum GLIPR 1.5 -------------------------------------------------------------------------------VDIAFGLWLNESRNFNFSSQS**C**-LKGQ**C**------KHYTQIVWENT------------------

>S. japonicum AllergenV5Tpx1.2 -------------------------------------------------------------------------------ITNAVDNWFNEYKLYDYKRNT**C**LKPKD**C**------LHYKRMVWGES------------------

>S. haematobium cA08597 -------------------------------------------------------------------------TAVHSELAEAVTYWMKESDHYDHKSDR**C**EPPHQ**C**------DSYKQIVQAET------------------

>S. mansoni Smp176180 -------------------------------------------------------------------------------VERAVKLWFSEAMYYNFNTNI**C**SSAT-**C**------GNYPQLVWENT------------------

>S. haematobium cC00745 -------------------------------------------------------------------------------VLVGFKMWFNEFQNYNYSSNT**C**PKG-Q**C**------THYTQLVWEET------------------

>S. mansoni Smp120670 -------------------------------------------------------------------------------LLIGFKMWFDESQNYNYNTNT**C**INN-Q**C**------THYTQLIWENT------------------

>S. haematobium cA08598 -------------------------------------------------------------------------------VEIGFQLWLNEYKNYDFFNRL**C**FTG-R**C**------SHYTQIVWENT------------------

>S. mansoni Smp070250 -------------------------------------------------------------------------------VEIGFQRWLNEYKNYDFFNRL**C**LVG-R**C**------SHYTQIVWENT------------------

>S. mansoni Smp179480 -------------------------------------------------------------------------------VDIGFQRWLNEYKNYDFFNRL**C**LVG-R**C**------IHYTQIVWENT------------------

>S. japonicum GLIPR 1.1 -------------------------------------------------------------------------------VKTGFQMWLDEYKNYDYYTRT**C**RMG-Q**C**------GHYTQIVWEDT------------------

>S. japonicum Allergen V5Tpx1.3 -------------------------------------------------------------------------------IETGFQLWLDEYKYHDFNTGT**C**HLA-Q**C**------THYTQIVWENT------------------

>S. haematobium cA09053 -------------------------------------------------------------------------------INTGFQLWLDEYKNYDFYTRT**C**RMG-Q**C**------GHYTQLVWEDT------------------

>S. mansoni Smp154260 -------------------------------------------------------------------------------IKTGFQLWLDEYNNYDFYTRT**C**RMG-Q**C**------GHYTQLVWEDT------------------

>S. mansoni Smp176160 -------------------------------------------------------------------------------IKTGFQLWLDEYKNYDFYTRT**C**RMG-Q**C**------GHYTQLVWEDT------------------

>S. mansoni Smp154290 -------------------------------------------------------------------------------IKTGFQSWLDEYKNYDFYTRT**C**RMG-Q**C**------GHYTQLVWEDT------------------

>S. mansoni Smp070240 -------------------------------------------------------------------------------VRQAASVWFEQHKNYNFENNT**C**EANKT**C**------ADYKQLAFADT------------------

>S. japonicum Pep. Inhibitor1.2 -------------------------------------------------------------------------------IRDAPAVWFNQHRNYNYTKNV**C**AAQKI**C**------ADYKQLAYAST------------------

>O. viverrini c7991 -------------------------------------------------------------------------------YTDAVRLWFEEYRFYDYRENT**C**ESGKL**C**------GHYTQLVWAET------------------

>O. viverrini c17638 -------------------------------------------------------------------------------LEAAYEAWFAEYKEYNYATRE**C**VGM--**C**------GHYTQAVWNEX------------------

>O. viverrini c18160 ----------------------------------------------------------------------------------AVNLWMAEHVDYNLEANT**C**THGKT**C**------LHYTQMVWASS------------------

>O. viverrini c15680 ------------------------------------------------------------------------------RIADPVESWYESRNVT-------------------GGYSQVV**C**ATT------------------

>C. sinensis c14383 -------------------------------------------------------------------------------YTDAVRLWFVEYRFYDYR---------------------------------------------

>S. haematobium cA08206 ----------------------------------------------------------------------------------------------------------------------ITFADT------------------

>F. gigantica c25411 -------------------------------------------------------------------------------VFDTVRLWF------------------------------------------------------

>C. sinensis c4741 ------------------------------------------------------------------------------LIGDPVQLWYEYRRKT-------------------EHYRKMVSPTA------------------

>O. viverrini c4477 ------------------------------------------------------------------------------VIGDPVQLWYAYRNKT-------------------GHYRKMVSPTA------------------

>C. sinensis c7351 ------------------------------------------------------------------------------AIGDPVELWYEYRYDT-------------------KHYDQVVSPDM------------------

>C. sinensis c5350 ------------------------------------------------------------------------------RIADPVESWYVGRKRT-------------------GHYSQVV**C**ATT------------------

>C. sinensis c13355 ------------------------------------------------------------------------------GQTDPVVTWFNEHENFTFGPTTNENLASV------GRYTQMVWSKT------------------

>O. viverrini c5997 ------------------------------------------------------------------------------AQIDPIEMWFNEQQNFKFGPITNENLATA------GRYTQMVWSKT------------------

>F. hepatica c6081 ------------------------------------------------------------------------------LSLDPLKEWFDEQSKYSYKPIDPSDFYLY------GHYTQMVWAET------------------

>S. japonicum PRP4 -------------------------------------------------------------------------------VDIGFGLWLNENIYYKFFNRS**C**LVGK-**C**------NHYTQIVSQNT------------------

>O. viverrini c3648 ----------------------------------------------------------------------------RGGVSDPVEMWYNEHWVYKMGQMRENETY------MVWEYIQIVTART------------------

>C. sinensis c10021 ----------------------------------------------------------------------------------AVRLWFEEYRFYDYRENA**C**EPGKL**C**------GHYTQLVWAETX-----------------

>S. haematobium cA06111 ----------------------------------------------------------------------------------------------------------------------------------------------

>O. viverrini c18119 ------------------------------------------------------------------------NVAIASSVERAVKLWAEEYVNYDFAKGKCKRGKQ**C------**LHYTQMAWASS------------------

>S. mansoni Smp176170 ----------------------------------------------------------------------------------------------------------------------------------------------

>O. viverrini c19644 ----------------------------------------------------------------------------------------------------------------------------------------------

**Group 3**

>O. viverrini c6176 ------------------------------------------------------------------------SQPTHIDGKQATLRWYGEITRFNFGEEKQRMS---------GNFSQIVWKDT------------------

>C. sinensis c2042 ------------------------------------------------------------------------SQPTHIDGKVNKLLFDG-----------------------------------------------------

>S. haematobium cA01987 ---------------------------------------------------------------------TTVAQKKDFT----------------------------------GHFTQVVWKST------------------

>S. mansoni Smp163400 ---------------------------------------------------------------------TAIAQKKDFTGADATKTWYQEIEDYDFKRENQFP**C**---------GHFTQVVWKST------------------

>S. japonicum GAPR 1.3 ---------------------------------------------------------------------STVAQKKDFTGVDVTKSWYQEIEDYDFKKENQFS**C**---------GHFTQVVWLST------------------

>S. mansoni Smp124060 ------------------------------------------------------------------------TGKAEMTGARATRNWYDEIHYHNFNKQFQSQS---------GHFTQLIWKNT------------------

>S. japonicum GAPR 1.1 TNKATMSDDLDFAGDPAFLSHTVTNAREDGQ**C**GISLYSNRPQRTLKEKEVLEAMEDYTYLGSSIINKQRGSDVNVKARIRSQATQNWYNEIHQHNFDQQYQSGT---------GHFTQVIWKST------------------

>F. hepatica c9426 ------------------------------------------------------------------------SGVAIMTGREATKMWYDEIILHDFKGGYNGAT---------GHFSQVIWKNT------------------

>S. haematobium cA00481 ------------------------------------------------------------------------TGKAELTGARATQNWYNEIHDHNFDKQFQSQT---------GHFTQVIWKNT------------------

>F. gigantica c1042 ------------------------------------------------------------------------SGVATMTGREATKMWYDEIILHDFKGGYNGAT---------GHFSQVIWKNT------------------

>S. mansoni Smp124070 ------------------------------------------------------------------------TAGASLNGREATRNWYDEIIQHDFNGQNQPGT---------GHFTQVIWKST------------------

>S. haematobium cA07983 ------------------------------------------------------------------------TAGASLNGREATRNWYDEIKQHDFNGQNQPGT---------GHFTQVIWKST------------------

>C. sinensis c3765 ------------------------------------------------------------------------EGRGPFGADEATKSWYDQGSMHDFGEGFTYET---------SYFSQLVWKDS------------------

>F. hepatica c10643 ------------------------------------------------------------------------TSEIFFSGADASKMWYMQSEFHDYNGEMTYES---------**C**YFTQMIWKDT------------------

>O. viverrini c8913 ------------------------------------------------------------------------EGRGPFGADEATKSWYDQSSMYDFGEGFTYET---------SYFSQLVWKDS------------------

>F. hepatica c1472 ------------------------------------------------------------------------NIPTVITGKQATLRWYSEVQRFHYGEERQSMS---------GNFSQIIWKSS------------------

>F. gigantica c3 ------------------------------------------------------------------------NTPTVITGKQATLRWYSEVQRFHYGEERQSMS---------GNFSQIIWKSS------------------

>F. gigantica c4035 ------------------------------------------------------------------------SGYLGITGLEVVQQWYKEIKHFRFGEEKQKN**C**---------NEFSQMIWKGT------------------

>S. haematobium cA03186 --------------------------------------------------VAP**C**QITDYNEALPPTLQLFSPHHSVSSMSEEVSQTWYSEIDYHDFNQ**C**YHPNS---------RHFTQMIWKST------------------

>S. mansoni Smp124050.1 ------------------------------------------------------------------------ASP**C**QITGEEVSQTWYSEIDYHDFTQSYHPNS---------RHFTQMIWKST------------------

>S. mansoni Smp124050.2 ------------------------------------------------------------------------ASPCQITGEEVSQTWYSEIDYHDFTQSYHPNS---------RHFTQMIWKST------------------

>S. mansoni Smp124050.3 ------------------------------------------------------------------------ASPCQITGEEVSQTWYSEIDYHDFTQSYHPNS---------RHFTQMIWKST------------------

>S. mansoni Smp124050.4 ------------------------------------------------------------------------ASPCQITGEEVSQTWYSEIDYHDFTQSYHPNS---------RHFTQMIWKST------------------

>S. japonicum GAPR 1.2 ------------------------------------------------------------------------AAP**C**QIT----------------------------------GHFTQMIWKST------------------

>C. sinensis c3166 ------------------------------------------------------------------------SVKAALSGQQATQMWYDEIHVHQYVEQFQPQS---------GHFTQVIWKGT------------------

>O. viverrini c4390 ------------------------------------------------------------------------SVKAALSGQQATQMWYDEIHVHQYVEQFQPQS---------GHFTQVIWKGT------------------

>O. viverrini c18440 ------------------------------------------------------------------------SQKATLTGSDASKMWYDEIKLHDFRGEFNEKS---------GHFTQLIWKST------------------

>F. gigantica c3823 ------------------------------------------------------------------------SSQGALSGKEATQMWYDEIQYHNFKGQFQPKS---------GHFTQVIWKSA------------------

>O. viverrini c11939 ------------------------------------------------------------------------ENQTTLTGKQATLMWYREIASYEFGVENQLN**C**---------GHFSQVVWKST------------------

>F. hepatica c9115 --------------------------------------------------------------------------IRRSSTSASVKKNKKIVTNFPKNSGKEHEEPVSAVPVYHT-DVQSLSSVS------------------

>S. haematobium cA00727 --------------------------------------------------------------------------------------------QKIDHLI**C**GQNMALII-----GYFTQMIWKKT------------------

>C. sinensis c1770 --------------------------------------------------------------------------RPSVSAERIVNEWYSEIRSHTFGAEKQPNS---------HNFSQLIWKST------------------

>O. viverrini c3766 --------------------------------------------------------------------------RPSVSAERIVNEWYSEIRSHTFGAEKQPNS---------HNFSQLIWKST------------------

>O. viverrini c11437 -------------------------------------------------------------------------E**C**GKITGQTLVEKWYKESELYDYSTEPNSVEYV-------GHFTQMIWKGS------------------

>F. gigantica c4654 ------------------------------------------------------------------------TAAVHFGAQDLVDHWYQE**C**SKYKFDTEPSSIQGI-------GGFTQVVWSGS------------------

>F. gigantica c12544 -----------------------------------------------------------------------TASHVDIQGTEVVNQWYADMKNYNFEAEKGPA----------GNFTQLVWSAT------------------

>S. haematobium cB00359 -------------------------------------------------------------------------------GHEVVNTWHSDAENYNYENGKGPA----------GNFTQLVWSST------------------

>C. sinensis c8455 ------------------------------------------------------------------------WENATITGEEATKSWYAQGDYHDFNESFTYET---------SYFSQLIWKGS------------------

>O. viverrini c2349 ------------------------------------------------------------------------WENATITAEEATKSWYAQGDYHDFNEGFTYET---------SYFSQLIWKGS------------------

>C. sinensis c14468 ---------------------------------------------------------------------------GKITGQTLVEKWYKESELYDYSTEPNSVEYV-------GHFTQMIWKGS------------------

>C. sinensis c13576 -------------------------------------------------------------------ATSISSQKATLTGSDASKMWYDEIKLHDFSGEFNGKS---------GHFTQLIWKST------------------

>F. gigantica c12861 --------------------------------------------------------------------------EIFFSGADASKMWYMQSEFHDYNGEMTYES---------**C**YFTQMIWKDT------------------

>C. sinensis c6993 ------------------------------------------------------------------------------------------------------------------HFTQVVWKDT------------------

**Group 4**

>S. haematobium cA03835 -------------------------------------------------------------------------------IKSIFKVWSTK--HYPYS**C**IVYD**C**----------SSYKQLVTYDT------------------

>S. haematobium cA06788 ----------------------------------------------------------------------------------------------------------------------------------------------

>S. mansoni Smp035980 -------------------------------------------------------------------------------INDAINVWRDGSRFYSLKNDE**C**KANYD-------**C**SMYKQAGGKAG-----------------

>S. mansoni Smp131370 -------------------------------------------------------------------------------NKSIFKVWSAKYDHYN--**C**IVMLTYD------QELADAARKWALE------------------

>S. haematobium cA07187 ----------------------------------------------------------------------------------------------------------------------------------------------

>O. viverrini c19841 ---------------------------------------------------------------------VGQNAAFASSVEQAVNLWMA-----------------------------------------------------

>S. japonicum Allergen V5Tpx1.1 ------------------------------------------------------------------------------------------------------------MIYENRAFHTSDWKST------------------

>S. haematobium cA00413 ------------------------------------------------------------------SVGQTVAEHTTIQKADDERPYEKDSN--------------------------------------------------

>C. sinensis c2194 ----------------------------------------------------------------------------------------------------------------------------------------------

**Group 1**

|-------| CAP2

>S. japonicum GLIPR 1.4 THVG**C**AVTR**C**KKTAKFPYG------VFVV**C**NYGPGASFKKSPYEVDSYEK-**C**PKTQSRWQNVNLNGNN**C**Y**C**YKN----------------------------------------------------------------

>S. mansoni Smp002070 EEIG**C**GYEK**C**GKN------------YLIV**C**NYAPGDSEDRPYEAKPES--K**C**NKSEN---------------------------------------------------------------------------------

>O. viverrini c962 TKLG**C**GATD**C**RGKNPDWKYG-----YYVV**C**NYRPAGNIRGRRPYVAGSRSD**C**VSASWSLWNKRGPLAVMYVFFLVSLSA-----------------------------------------------------------

>C. sinensis c4257 THIG**C**AVGN**C**KETPVL-SAN-----FVTV**C**FYAPYTHDKHRPVYEFGTEED**C**IPNKSGNSLVLSVFSKGSFWKINN**C**IVFDLVFL**C**FFAPR-----------------------------------------------

>F. hepatica c6577 VSIG**C**AYNR**C**NRTADFPYD------LAVV**C**NYGPQANFSTQPYEKGEKT-M**C**SGTTHSVFQWINRTQLLAIILWWLNNFFSLMNLISLIF**C**TPLLLNNTVSSRPLFFARKGIIAATR**C**SFQSLX--------------

>S. mansoni Smp193710 AYVG**C**GFTR**C**RQYEYPS-N------MLIA**C**YYSPKVKSGPPYTDATNRR--**C**KYLNANLNKIKGKGKN----------------------------------------------------------------------

>S. haematobium cA06447 TEIG**C**GVQK**C**GQR------------FLVV**C**NYSPAAEEDRQPYEKGTQE-D**C**DDVDDAEYN-----------------------------------------------------------------------------

>S. mansoni Smp123090 KHIG**C**GLNK**C**KKRK-----------LMIV**C**NYSPSADDKGKPYVIGDRSQ-**C**LLNEN---------------------------------------------------------------------------------

>S. haematobium cA06474 EFIG**C**STGY**C**SGLTEVKSG------KIIV**C**YYSPMSDVQASRPYEENKIDP**C**PHPPPITTTTTTTTTRKPITVTRKLQKGPYGRYR**C**N**C**K**C**QPN--------------------------------------------

>S. mansoni Smp001890 TEIG**C**GVQK**C**SNR------------FLVV**C**NYSPAAEEDKQPYEKGTQE-N**C**DDVDDAEYN-----------------------------------------------------------------------------

>C. sinensis c1247 TKLG**C**GATD**C**RGKNPDWKYG-----YSVV**C**NYRPAGNLRGRRPYVAGTRSD**C**VSASWSLWNKKGPVAVTHIFFLVSLSITKYLISV**C**NKKVQSNNAHVYRNTRLFLKKX-----------------------------

>S. jap Pep.inhibitor1.3 KHIG**C**GAKW**C**SDFKPPLK-------YLVV**C**NYSPALGNQLEMTRDENLLLYYHNYIRRERNT**C**DYHTSIPADVKLNDYVSN---------------------------------------------------------

>S. mansoni Smp127130 EFIG**C**SVGH**C**NTHTEVKSG------QIIV**C**YYSPMGDVKTSQPYVENKIDP**C**RHPPITTTTTTKPITVTRKLQMGPYGRYR**C**D**C**K**C**QPN-------------------------------------------------

>S. mansoni Smp123550 THVG**C**GITN**C**TGSYSFPYG------LSVV**C**NYGPGGNYEGRYPYEAKSQDE**C**YATTTKRPTTTKRPTTTKRPSTTTTKRPGTTPTQKPGVPKQIPKPIWPSIISTWNEYATSNMIQGIVTQT**C**I**C**VKN----------

>S. japonicum GLIPR 1.3 THVG**C**GVTN**C**TGYYGFPYG------LSVV**C**NYGPGGNYEGRQPYETKTEEE**C**HAVTTMKPTTRPRTTRRPGTPKKLPKPDWTTLIPTWRDFATSNHLTGIVTQT**C**I**C**IDN----------------------------

>S. mansoni Smp123540 THIG**C**GVTD**C**RKSPQFPYG------VFVV**C**NYAPGAKFDKSPYDVVSHA-K**C**SILELKWKRFNLNGQN**C**Y**C**YLN----------------------------------------------------------------

>S. japonicum GLIPR 1.2 TDIG**C**GVAK**C**PEYK----G------LSIV**C**NYGPAGNWNNEKPYEVKRHDE**C**PTVHKWASHNTQRTPVNRNADAFVRDRNQRNMPVSSEIRQPRIRIRHFDKFSKTPIE**C**R**C**YPVNERLN------------------

>F. gigantica c566 RAVG**C**AFKD**C**SNIRNFPYG------KSIA**C**NYGPAGNQEGRRPYVKGDEKG**C**TSINKTNKNDQLMRVVLVRALVIYDLFNFSLILLYYMKIALHFGRIIVSSRISF**C**NARLLNFPIHSLIDIIKRYLEX---------

>F. gigantica c7108 VSIG**C**AYNR**C**SRTADFPYD------LAVV**C**NYGPQANFSTQPYEKGEKTI-**C**SGTTHSVFQWINRIQLLAIILWWLNNFF----------------------------------------------------------

>O. viverrini c4326 THVG**C**AVGN**C**RDNPVSSAN------FVTI**C**FYAPYQPDKDRPIYEIGTEED**C**IPNKSGNSLVLLVTSNESFSKNIQLQ**C**SGPNKX-----------------------------------------------------

>F. hepatica c4375 THIG**C**AFKE**C**PKTAQFQYG------KSIV**C**NYGPAGNFNNEKPYVLGAQSI**C**PPATNTTTTTTIITATLNNISDLPRQEIWIQLGVI**C**GF**C**VIELLRLMNF**C**TYPINIHPNISVEQWILQFVQHI**C**ATINSLNLLA**C**F ...

>S. haematobium cA08594 AYVG**C**GYSR**C**EGVGYPNE-------KLIT**C**FYSPAVRNGQPYTDGTNGR--**C**KI------------------------------------------------------------------------------------

>S. mansoni Smp160250 AYVG**C**GFTR**C**RQYEYPSS-------MLIA**C**YYSPKVKSGPPYTDATNRR--**C**KI------------------------------------------------------------------------------------

>S. mansoni Smp159280 TDIG**C**GVAN**C**PRYG-----------LSIV**C**NYGPGGNWNNEKPYEVKPRHM**C**SMMQNIPQNSLQTNSAHTQHGQKPIKKSEERVSRN MQYGRRIRDLRVYPK**C**RDN-------------------------------

>S. mansoni Smp159290 TDIG**C**GVAN**C**PRYG-----------LSIV**C**NYGPGGNWNNEKPYEVKPRHM**C**SMMQNIPQNSLQTNSAHTQHGQKPIKKSEERVSRNMQYGRRIRDLRVYPK**C**RDN--------------------------------

>S. mansoni Smp002630 TDIG**C**GVAN**C**PRYG-----------LSIV**C**NYGPGGNFNNEKPYEVKPRNM**C**PKVQNIPKNSLQTSRAHTQHVQKPIKQSQKKVSWIVQNGRKDRNQRKQSRSRYN--------------------------------

>S. mansoni Smp193680 TDIG**C**GVAN**C**PRYG-----------LSIV**C**NYGPG-------------------------------------------------------------------------------------------------------

>S. mansoni Smp078490 TDIG**C**GVAN**C**QRYG-----------LSVV**C**YYGPGGNWINEKPYKVKPHNL**C**PIVQSIPKDHFQTNRVHTRHGQKSINLREKKGVKYRPKN-----------------------------------------------

>S. mansoni Smp139450 TDIG**C**GVAN**C**SMYG-----------LSIV**C**NYGPGGNWINEKPYEVKPHNL**C**PIVQNISKDSFPTNRAHTRRGQKSLKQIEKKGVKYHSNN-----------------------------------------------

>S. mansoni Smp141550 AYVG**C**GYTR**C**EEYEYPSN-------MLIA**C**YYSPKVMSGPPYTDGTNGR--**C**GSEN----------------------------------------------------------------------------------

>S. mansoni Smp141560 AYVG**C**GYTR**C**EEYEYPSN-------MLVA**C**YYSPKMMSGSPYTDGTNGR--**C**GSEN----------------------------------------------------------------------------------

>S. japonicum expressed protein ------------------------------------------------------IGEYETIGQNIAEYPTIEQLVIIINVYIILIRKLYQSFN---------------------------------------------

>F. hepatica c8831 --------------------------------------------------------------------------------------------------------TSSWKX----------------------------

>S. haematobium cA08595 AYVG**C**GYSR**C**EGVGYPNE-------KLIT**C**FYSPAVRNGQPYTDGTNGR**C**----------------------------------------------------------------------------------------

>C. sinensis c1246 THIG**C**GVRD**C**RDTGSFPYG------LSIV**C**NYGPAGNFVGAKPYEEGSSAD**C**KPVGETNSPKPPDEGDQGVELPTESGQEAEQPGENDQGAVPPSETPPPQRPPSPQGSRPHQGSWPPQGTRPHHGSWPSQGSWAPQG ...

>O. viverrini c2686 THIG**C**GVRD**C**RDTGSFPYG------LSIV**C**NYGPAGNFVGAKPYEEGSSAD**C**KPAGGTDSSKPPGESDQGEELPTEGGQGAELPGESDQGGAPPDDQNEVPPPQDTEPPIETLPPQGSRPPHGSRP------------

>S. haematobium cA08596 AYVG**C**GYSR**C**EGVGYPNE-------KLIT**C**FYSPAVRNGQPYTDGTNGR**C**----------------------------------------------------------------------------------------

>S. japonicum Pep.inhibitor1.1 THIG**C**GVAK**C**PEYQG----------LSIV**C**NYGPGGDWNNEKPYIVKPPDE**C**PKFQDVVSHNNHSSSVDHNPDASGSDRTHQNMSMSSNIRDAS**C**TLKLSIKSLKGLIE**C**R**C**YPVNERRN------------------

>C. sinensis c2194 TKLG**C**GVKN**C**PNIGG----------TLYV**C**DYKPPGNYWGAKPYTAGTKED**C**LKSTTSPPNSKPSGDGGMVGKPATDSTSTGGNLRQTSKMVAAAELIFILPVVFNAWHLTFSKKNTSKVKK**C**GQN------------

>C. sinensis c353 THIG**C**GFAR**C**PSQPWR---------HAFV**C**NYGPAGNMRMRTLNGAIIVLPPYEESST**C**PGHLKNLMNTFKLEIFNIVNSHYMYS**C**KHPFGPFRWAKPQDYYRDFLSK------------------------------

>O. viverrini c1174 THIG**C**GYAE**C**PPQPWH---------HIFI**C**NYGPGNVKFHGDLLDNTLMSLKFSRLYVT**C**HFLRL**C**KPQERNYPWPX-------------------------------------------------------------

>O. viverrini c15215 ------------------------------------------------------------------------------------------------------------------------------------------

>S. haematobium cA01429 ------------------------------------------------------------------------------------------------------------------------------------------

>O. viverrini c6515 TKLG**C**GVKH**C**PENRT----------TLYV**C**DYKPPGNYLGQKPYTAGTEDD**C**LKSTTSPPPTSPPANPPTGRLSENAETVGKPELESARNKGSTTGSTSVGRNLWQAREMAAAKLIFSLTGCFVSMISYFLEIINNQ ...

>F. hepatica c8830 RAVG**C**AFKD**C**SDIPNFPYG------KSIA**C**NYGPAGNREGMRPYVKGDEKS**C**TSVNKTSKNEQLMRV-----------------------------------------------------------------------

>S. mansoni Smp116210 TDIG**C**GVAN**C**PRYG-----------LSIV**C**NYGPGGNWNNEKPYEVKPRHM**C**SMMQNIPQNSLQTNSAHTQHGQKPIKKSEERVSRNMQYGRRIRDLRVYPK**C**RDN--------------------------------

>S. mansoni Smp118160 TDIG**C**GVAN**C**PRYG-----------LSIV**C**NYGPGGNFNNEKPYEVKPRNM**C**PKVQNIPKNSLQTSRAHTQHVQKPIKQSQKKVSWIVQNGRKDRNQRKQSRSRYN--------------------------------

>S. mansoni Smp120240 TDIG**C**GVAN**C**PRYG-----------LSIV**C**NYGPGGNWNNEKPYEMKPRNL**C**PKVQNIPKNSLQTNSAHTQHGPKPLTQSEERVSTNVQNGRRE**C**NQREQLRSRYN--------------------------------

>S. mansoni Smp100560 TDIG**C**GVAN**C**PRYG-----------LSIV**C**NYGPGGNWNNEKPYEVKPRHM**C**SMMQNIPKNSLQTNSAHTQHEQKPLTQSEEKVSTNVQNGRRE**C**NQREQSRSRYN--------------------------------

**Group 2**

>C. sinensis c3048 TDLG**C**YTHL**C**PLITSIGGMRWTNA-YYTI**C**KYAPPASLLDPRPYE---------------------------------------------------------------------------------------------

>S. mansoni Smp002060 TDIG**C**ASNK**C**GNR------------YMVV**C**NYAPGADDERPYEKDSN-------------------------------------------------------------------------------------------

>S. japonicum GLIPR 1.5 THIG**C**GVAT**C**KNSPFT---------LSIV**C**NYGPGGNLIGQVPYRLKIENKLIKPEIQN**C**NVRKYTYPLITFVPRLGPSLIKPLHSPNVSRI----------------------------------------------

>S. jap AllergenV5Tpx1.2 EFIG**C**SVGR**C**PQPEIQTG-------QIVV**C**YYSPMTSVKDSRPYEEDIYNP**C**PPPQPETTTIIVPTTKKIVITTERLKMGPYGRYR**C**K**C**T**C**KPN--------------------------------------------

>S. haematobium cA08597 AYVG**C**GYTR**C**PGYEYPAN-------MLIA**C**FYSPAVIKRQPYTDEKN**C**G**C**KNVTEGQNGINEKKK-------------------------------------------------------------------------

>S. mansoni Smp176180 TDVG**C**GVTD**C**PNFRTK---------LVIV**C**NYGPGGNIPEQRPYRTAN------------------------------------------------------------------------------------------

>S. haematobium cC00745 TDFG**C**GVTV**C**KNMTPS---------LNVV**C**NYGP--------------------------------------------------------------------------------------------------------

>S. mansoni Smp120670 TDFG**C**GVTE**C**KNMLPN---------LNVI**C**NYGPG-------------------------------------------------------------------------------------------------------

>S. haematobium cA08598 TDIG**C**GVTS**C**PHSPFN---------LSIV**C**NYGPAGG**C**PGHFPYDVKGQYRQSNIRYGRQWYNRRYGR**C**RRAYVKQK**C**NLENAKRLNQTPEIQNRKYKLIKQYL**C**PNK------------------------------

>S. mansoni Smp070250 TDIG**C**GVAT**C**PHSPFK---------LSIV**C**NYGPGGG**C**PRQFPYSVKGLYRQWTIKYGRKWYSWRYGRR**C**HREYVKQR**C**NHTNERQLNQTPVIQTKGYKLIKQYL**C**PNKQKVN-------------------------

>S. mansoni Smp179480 TDIG**C**GVAT**C**PHSPFK---------LSIV**C**NYGPGGG**C**PRQFPYSVKGLYRQWTIKYGRKWYSWRYGRR**C**RPVYVKQR**C**NHTNERQLNRTPAIQKKRYKLIKQYL**C**PNKQKVN-------------------------

>S. japonicum GLIPR 1.1 TDVG**C**GVTD**C**PNFIYG---------LSIV**C**NYGPGGNYAGREPYKIAN------------------------------------------------------------------------------------------

>S. japonicum Allergen V5Tpx1.3 TDIG**C**GVSN**C**PNIPYK---------LSIV**C**NYGPAGNYIGQAPYKTATGVTDSEPQQQQTKQTVTSGYNSNNNSSNNNSNNNTSSNNNTSDISDNNSSNSNSNNDIINNNNNNNNNNNNNGNSNNDINSSNNNISSST ...

>S. haematobium cA09053 TDVG**C**GVTN**C**PNFPYG---------LSIV**C**NYGPGGNYVGRSPYRT--------------------------------------------------------------------------------------------

>S. mansoni Smp154260 TDVG**C**GVTD**C**PNFPYG---------LSIV**C**NYGPGGNYPGRPLYRTTN------------------------------------------------------------------------------------------

>S. mansoni Smp176160 TDVG**C**GVTD**C**PNFPYG---------LSIV**C**NYGPGGNYPGRPLYRTTN------------------------------------------------------------------------------------------

>S. mansoni Smp154290 TDVG**C**GVTK**C**PNFPYG---------LSIV**C**NYGPGGNYAGRPLYRTTN------------------------------------------------------------------------------------------

>S. mansoni Smp070240 THIG**C**GYAM**C**FNLTGLDK-------VFVV**C**NYGPGGKYANRQPYDPIYPEDPYYLPN---------------------------------------------------------------------------------

>S. japonicum Pep. Inhibitor1.2 THIG**C**AYKF**C**EKLNGTGK-------ILVV**C**NYGPGGKFINRKPYQIFDYDDFYLYN----------------------------------------------------------------------------------

>O. viverrini c7991 RKIG**C**GVQN**C**PASTFP--------------------------------------------------------------------------------------------------------------------------

>O. viverrini c17638 ------------------------------------------------------------------------------------------------------------------------------------------

>O. viverrini c18160 TLLG**C**GVTE**C**PKNGT----------TVFI**C**DYKPPGNYEGARPYEAGTQAD**C**VTSSTSPPTITPTTAGQPDEDVTPESSMX---------------------------------------------------------

>O. viverrini c15680 RRVG**C**IMVS**C**PKLVIPSANRTELNAFFSV**C**HYWPPKHANQEPYEKX--------------------------------------------------------------------------------------------

>C. sinensis c14383 ------------------------------------------------------------------------------------------------------------------------------------------

>F. gigantica c25411 ------------------------------------------------------------------------------------------------------------------------------------------

>C. sinensis c4741 VYLG**C**YMNR**C**DVLQLVAAGVNQTHAYYTV**C**RYSTTKTTPKVPTENMNLLDPSVNANVVLLPKAVWTNSR**C**TMFVNNVTLL**C**VFILNFVVVVVVVVXXXX---------------------------------------

>O. viverrini c4477 VYLG**C**HMTR**C**AVLQLVQAGVNQTNAYYTV**C**RYSTIKTMPTENMNLLDPPVNARSFILAEHGMNELTLQYV**C**LK**C**ASVLRLYFLNFASQPRNGKRTVLS----------------------------------------

>C. sinensis c7351 VYLG**C**HTAF**C**PKLQVVKSNSVATNAYYSV**C**RYSYQRMQNTGPRKANNFFNQFQPNL**C**KDFALILMLFY----------------------------------------------------------------------

>C. sinensis c5350 RQVG**C**IMIT**C**PTILIPEQNETLSNVFYSV**C**RYMPPIFVGQEPYEKVNNVKGKQNKNMTFYNMHLLI**C**FEFTTVFQLSKVNVX--------------------------------------------------------

>C. sinensis c13355 KELG**C**YQKL**C**VTLEAMGKTWKNA--YYTV**C**RYSPPGNILGTTPYTPIRDELQPSTVATTPTTTTTTTTX---------------------------------------------------------------------

>O. viverrini c5997 KEVG**C**YQKL**C**STVTAMGKTWQNA--YYTV**C**RYSPSGNLLG--------------------------------------------------------------------------------------------------

>F. hepatica c6081 TSVG**C**WRNQ**C**GYLTFPGGSPMYNA-YYTV**C**KYYPPGNYFGRLPYKRINLDRTNSANIGNTDETSRSVVDQYELFRSATIQX---------------------------------------------------------

>S. japonicum PRP4 THIG**C**GVAT**C**KNSPFK---------MSIV**C**NYGPGGNHTSEFPYRAKGIDKPVTPMLPKPEHHEPPTVNKSLPSLLKAPKLKSST-----------------------------------------------------

>O. viverrini c3648 TDLG**C**FMHL**C**PELTAPSGMKWTDA-YFTV**C**RYAPPANRLDPKPYPIAGDEIPTETTTIRTTTTPTTTTTPIIITTHPTTTMSTEDILDPA------------------------------------------------

>C. sinensis c10021 X-----------------------------------------------------------------------------------------------------------------------------------------

>S. haematobium cA06111 TDVG**C**GVTN**C**RNFKTK---------LVIV**C**NYGPGN------------------------------------------------------------------------------------------------------

>O. viverrini c18119 TSLG**C**GVKH**C**PKNGT----------TLYV**C**NYKPPGNYRGQKPYTAGTKKDCLTSTTVNGPKQKRSTTSGSGNLWQTRKTTX--------------------------------------------------------

>S. mansoni Smp176170 ------------------------------------------------------------------------------------------------------------------------------------------

>O. viverrini c19644 ------------------------------------------------------------------------------------------------------------------------------------------

**Group 3**

>O. viverrini c6176 RRVGFGRTIKDGG**C**----------KIYIVAYYTP**C**GNVTGHFTENVPLPLSGELYIPTDAEKGWNTDNDPLSVASSFTLLFSFYFVINLLTSFML**C**VTSLFX------------------------------------

>C. sinensis c2042 ------------------------------------------------------------------------------------------------------------------------------------------

>S. haematobium cA01987 TTAGFGRAWSKDRH----------SIYVVGRYDPPGNFSDEFLENVPPLIMNLRSDN---------------------------------------------------------------------------------

>S. mansoni Smp163400 ITAGFGRAWSKDRH----------SIYVVGRYDPPGNFSDEFLENVPPLIKRRKKATAMFNKRN--------------------------------------------------------------------------

>S. japonicum GAPR 1.3 ITAGFGRAFSKDLH----------SIYVVGRYDPPGNFSDEFSENVPPVITNNQ------------------------------------------------------------------------------------

>S. mansoni Smp124060 SKAGFGIQHSVDGH----------HVFIVGRYEPPGNVNGQFLENVPPPIHGQSTPKSKVPSYKHNEQNGPRRTYQDELVIVRETDRKDHNGSNHITLIDSSKRSRSDETIPNKNEVRIIRAEKKRQRR**C**AKR**C**SIMN

>S. japonicum GAPR 1.1 TKAGFGIQHSTDGH----------HVFIVGRYVQLGMYKVNSKKMYQDRIHVEYQLLTYVKYDRRSSDASGVRHLSPMTTNDSHFMSQINESANSTIESLVAQWYRWLSVHSKSKVSSQNRDTSNVNGPQRNNRAKSK ...

>F. hepatica c9426 TNAGFGRASTKDGH----------KMFMVGHYTPPGNVQGEFEANVPRLTSRKKSSVFGKLFQKRKKSKEPKVVSKKHGKNSAGEKEIVVNSKKRGKSQSTEREIVVPPNSHSRKSASPAKQTLFSPSTTSSSTHRVV ...

>S. haematobium cA00481 SKAGFGIQYSNDGH----------HVFVVGRYEPAGNVYGEFQENIPRPIHGQSTPKSKEFSYQHDEPNGPRRT**C**RGRFD**C**EINNFPFVN------------------------------------------------

>F. gigantica c1042 TNAGFGRASTKDGH----------KMFMVGHYTPPGNVQGEFEANVPRLTSRKKSSVFGKLFQKRRKSKEPKVVSKKHEKNSAGEKEIVV------------------------------------------------

>S. mansoni Smp124070 IKAGFGSALSKDGK----------KVYVVGRYKPAGNIIDLYEDNVPKPKITAPPRDEFIKIPESK**C**SILN-------------------------------------------------------------------

>S. haematobium cA07983 NKAGFG------------------------------------------------------------------------------------------------------------------------------------

>C. sinensis c3765 KLVGFGRATSSDGT----------ASYIVAHYSPKGNIRDRFHENVSYASRPLTHSSLQELRPPEITSRPLSTLSSKELKEREKQEKKLRERQEKERKEMEKRMKKDLKQREKLEKKERRKSKANVERQR**C**IAFLGQL ...

>F. hepatica c10643 KIVGFGQATSQDGY----------ASYIVAHYMPKGNVRGQFSFNVPPARLDAFAEQYDPFHRPITQTLPAVSQPTTPTVLPSLPETKEERKAREKAEKKERERLERERKETEKREKKARKELEKIEKKEKRKSLKLN ...

>O. viverrini c8913 KLVGFGRATSSDGT----------ASYIVAHYSPKGNIRDRFHENVPYASRRLTHSSLQELRPPEISSRPVSTLSSKELKEREKQEKKLRERQEKERKELEKRIKKEQKQREKLEKKX--------------------

>F. hepatica c1472 THAGFGLTTKNGG**C-**---------NIFIAAYYRPSGNVSGHFTENVPRPLNGVDYLPSNKEMGWNHTRMYPPWYLLNSIHPSQQQNFISSFNLLHFVFTESIQ**C**FTSSKDITFILLLPQSYHQLALLQENKTX-----

>F. gigantica c3 THAGFGLTTKNGG**C-**---------NIFIAAYYRPSGNVSGHFTENVPRPLNGVDYLPSNKEMGWNHTRMYPPWYLLLSIHPSQQQNLISSFNLLHFIFTEFIQ**C**FASSKDITFILSLPQSYHLLALLQDRKSGSAGMP

>F. gigantica c4035 RRAGFGRASLPHG**C**----------AIFVVGFYMDRGNVEGGYTENVPPLIETKAILPFDELLIKQL**C**NFKQAFIFDLESPFITHSFHSDAFIASLILLISRNTNNVNFINIHHT**C**QX---------------------

>S. haematobium cA03186 THAGFGLALSQDQT----------KAYVVGRYLPVGNRGDFGWNVPHYQGVKRSASPNQYLNENN-------------------------------------------------------------------------

>S. mansoni Smp124050.1 TRAGFGLAFSQDQT----------KAYVVGRYLPVGNKGDFGWNVPHYQGIKRSGDNESR**C**TGDGGSTKLNDTGSANNDTSRESMYSLDRNSIRSSHRRSFRESLGLSER**C**K**C**YWDHTPYAPEGHSLINEDERSRTGS ...

>S. mansoni Smp124050.2 TRAGFGLAFSQDQT----------KAYVVGRYLPVGNKGDFGWNVPHYQGIKRSGDNESRCTGDGGSTKLNDTGSANNDTSRESMYSLDRNSIRSSHRRSFRESLGLSERCKCYWDHTPYAPEGHSLINEDERSRTGS ...

>S. mansoni Smp124050.3 TRAGFGLAFSQDQT----------KAYVVGRYLPVGNKGDFGWNVPHYQGIKRSGDNESRCTGDGGSTKLNDTGSANNDTSRESMYSLDRNSIRSSHRRSFRESLGLSERCKCYWDHTPYAPEGHSLINEDERSRTGS ...

>S. mansoni Smp124050.4 TRAGFGLAFSQDQT----------KAYVVGRYLPVGNKGDFGWNVPHYQGIKRSGDNESRCTGDGGSTKLNDTGSANNDTSRESMYSLDRNSIRSSHRRSFRESLGLSERCKCYWDHTPYAPEGHSLINEDERSRTGS ...

>S. japonicum GAPR 1.2 THAGFGLALSRDKT----------KAYVVGRYLPVGNIGDFGWNVPHFQGVRRAGKN---------------------------------------------------------------------------------

>C. sinensis c3166 QKAGFGRASSSDGK----------SIYVVGRYTPPGNMVGKF**C**ENVPRPIRPVEKNTFNLSIDLVFGLNTRSNIDAG-------------------------------------------------------------

>O. viverrini c4390 QKAGFGRASSSDGK----------SIYVVGRYTPPGNMIGKF**C**ENVPRPIRPVEKVPAKKRRKRAK**C**VIQNKPSLHRKAVFLSFNAQSIWFQTDSEEVAQLPMPSHGSKSNFSFSLFY**C**SQRPVIF**C**IAHDTQYVRLS ...

>O. viverrini c18440 VKAGFGVASTKDGH----------QVFVVGRYMPPGNMLGEFFKNVPPVLTA GAAQFNPKHILPTRPRPKIIMHGNFRRKRERHTAPSIANIVNNNTY**C**Y**C**LSINNLF**C**PNFYLVN**CC**FDAQYFLNNIPKYLLSYFS ...

>F. gigantica c3823 KKAGFGRAVSADGK----------AVYVVGRYTPAGNVQGQFTENVPKSTKPVKPISDDPVAIDDLANKETITLPNNRRRRKRGL-----------------------------------------------------

>O. viverrini c11939 THAGFGRALKPDGR----------RIFVVGIYLPPANFNNEWTENVPAPLSGVIYTPTLEDIEEIPVSQTIGSRG---------------------------------------------------------------

>F. hepatica c9115 ------------------------------------------------------------------------------------------------------------------------------------------

>S. haematobium cA00727 KKVGFGFTKSEIGN----------IIFVVGHYLPAGNKTTEFQDNVLPRREGAHDMKTDDD**C**SNDPNENLKQHRHS**C**SKGRSFVKVLF**C**E**C**IVEEN------------------------------------------

>C. sinensis c1770 TRVGYGRTTIHGG**C**----------TAFVVALFQARGNVTGDYTENVPPRLDGTIPVDLGSAGLIIMNTWFNNYTNQTILWYFNFSLVFGESIFLHLWKHLNSKIYTNRLSAA--------------------------

>O. viverrini c3766 TRVGYGRTAIHGG**C**----------TAFVVALFQARGNVPGDYTENVPPRLDGTIPVDLGSAGLIIMNTWFRNYTKQILLWYFNFAFVF**C**ENIQLPTTPPVETFEFKNTHVTFASGTFR--------------------

>O. viverrini c11437 KEIGVGIAPSRDLPNR---------AFIV**C**FYNPPGNAKGEYRANVX-------------------------------------------------------------------------------------------

>F. gigantica c4654 QRIGVGRAMQPKSGPGGSTSGPGYKMVAV**C**FYYPPGNVTGQFKTNVKPSSRNYFPNRWNPYVNTSRYMELPHL**C**FVSLFIAVSMPLIHINDEHLHLHILDTQFLSVVIISQYSVIHLPLQSTSPPLLSITLVL**C**HVHL ...

>F. gigantica c12544 REVGFGKARAPGK------------**C**IVVAHYRPPGNVRGHYAENVHPPTGGLPSGGGHSPTGSVQQAPGTRKTVVTENVTDP-------------------------------------------------------

>S. haematobium cB00359 REVGFGKA**C**GPGK------------**C**VVVAHYRPPGNVLGRYLENVFRPKESVKEGVKQPIRNTFALNN---------------------------------------------------------------------

>C. sinensis c8455 KNVGFGRAVSEDGE----------AAYIVAHYFPKGNIRSVFSNNVPKL**C**SAPSANTTGTP-----------------------------------------------------------------------------

>O. viverrini c2349 KNVGFGRAVSEDGE----------AAYIVAHYFPKGNIRSVFSNNVPKL**C**SAPSANAIGTPVSTPNMRYTKLETKKELKEREKAEKKARERAEKERKEREKQLKKEQKEREKQAKKDKQKSKSLSGINKFESFNTFTA ...

>C. sinensis c14468 KEIGVGIAPSRDLP----------------------------------------------------------------------------------------------------------------------------

>C. sinensis c13576 VKAGFGVASTKDGH---------QIFIVGRYMPPGNMLGEYLKNVPPPVAADSAQLSTKHILPTRPRPKIIMHGNFRRKRDRHTAPSIAVVNNNY**C**H**CC**QINMDFFARIFNNNTSVSTHNVFYEISX-----------

>F. gigantica c12861 KIVGFGQATSSDGY----------ASYIVAHYMPKGNVRGQFSFNVPPARLDTFAEQYDPFHSPITRTLPAVSQPTTPTVLPSLPETKEERKAREKAEKKERERLERERKETEKREKKARKELEKIEKKEKRKSLKLN ...

>C. sinensis c6993 KKAGFGRAYTKDGR---------KIYVVGRYYPPGNYQGSCKLNVPRPISNGCYVSX---------------------------------------------------------------------------------

**Group 4**

>S. haematobium cA03835 RNIA**C**SIGD**C**KKQSNSGN--------IIL**C**NYYPVVSVNNRPDEAYKDESNTNSYTVTIEVPPETTSYMLDRGR**C**E**C**I**C**N----------------------------------------------------------

>S. haematobium cA06788 PEGHSIINEDERSRTGSIRSGDFQRSRPTSFSNNDDIHFKSVYTRSSEDLRPKTINVTTTSLPNTDDAHNSL**C**SPTVINLNFMNSDERPITNTKPSDDFSGSNRVSIRTSMRRPTRSSTPV**C**NNN**C**LTELYFAN----

>S. mansoni Smp035980 KDSGKAKAKAISRSHRAGLQFPVGRIHRHLKARTTSHGRVGATAAVYSAAILEYLTAEVLELAGNASKDLKVKRITPRHLQLAIRGDEELDTLIKATIAGGGVIPHIHKSLIGKKLPPPKPLSMN-------------

>S. mansoni Smp131370 ---------**C**RAERSSSAD----RVTKKWGLNSSLANADNRPDEVYENESNKNFYTVTTEVPPETTSYVLEHGR**C**E**C**I**C**N----------------------------------------------------------

>S. haematobium cA07187 ----------------------NGPMEHN**C**KDLIEHWYQESEKYKFNSEPDSIQGIVTTIKTYIFETSQNTVKTDPKKIGGLSKQMKTN-------------------------------------------------

>O. viverrini c19841 ------------------------------------------------------------------------------------------------------------------------------------------

>S. japonicum Allergen V5Tpx1.1 NKAGFGSAKSKDGM---------KVYVVGRYKPAGNVIGHYTDNVPRPKKNAPPSKKKTN------------------------------------------------------------------------------

>S. haematobium cA00413 ------------------------------------------------------------------------------------------------------------------------------------------

>C. sinensis c2194 ------------------------------------------------------------------------------------------------------------------------------------------
