# Supplementary material for: Insights into SCP/TAPS Proteins of Liver Flukes Based on Large-Scale Bioinformatic Analyses of Sequence Datasets
Source: PLoS One. 2012 Feb 22;7(2):e31164. doi: 10.1371/journal.pone.0031164 (PMC3284463; doi:10.1371/journal.pone.0031164)
Supplement: Figure S2 — Structure-based alignment of full-length amino acid sequences of putative double-domain SCP/TAPS proteins predicted from the transcriptomes of Clonorchis sinensis , Opisthorchis viverrini , Fasciola hepatica and Fasciola gigantica (liver flukes), and the genomes of Schistosoma mansoni , S. japonicum and S. haematobium (blood flukes). (DOC) [file pone.0031164.s002.doc]

**Supplementary Figure 2**. Structure-based alignment of full-length amino acid sequences of putative double-domain SCP/TAPS proteins predicted from the transcriptomes of *Clonorchis sinensis*, *Opisthorchis viverrini*, *Fasciola hepatica* and *Fasciola gigantica* (liver flukes), and the genomes of *Schistosoma mansoni*, *S. japonicum* and *S. haematobium* (blood flukes).

**Domain 1**

1

>C. elegans VAP1 MAVLAVVLLLA***C***LERAVAQTFG***C***SNTKINDQARKMFYDAHNDARRSMAKGLEPNK***C***GLL--

>S. mansoni Smp012350.1 -------------------------MSRLDDFRED***C***FRAHNEKRLLHGV------***C***A----

>S. mansoni Smp012350.2 -------------------------MSRLDDFRED***C***FRAHNEKRLLHGV------***C***A----

>S. japonicum GAPR-1 -------------------------MTRLDDFIED***C***LKEHNAKRLIHGV------***C***A----

>F. gigantica c6239 ---------------------------ERDALNTE***C***LLEHNRLRALHG-------***C***L----

>F. hepatica c10262 --KTLVPRWTADRQLPGSIILSAMSVTERDALNTE***C***LLEHNRLRALHG-------***C***L----

>S. haematobium cA07851 ---------------------MIYANGSLDDKSRELINLHEKYRQDLVN------***C***KVDGQ

>S. haematobium cA00818 ------MFNILIIYIIINYVY***C***VNINRKLDKNSEEILELHRKYRQDLVD------***C***KVDGQ

>C. sinensis c4469 ... -----------------SLTLFQLSMPNMDDFIEE***C***LREHNAKRELHGA------------

>O. viverrini c4948 -------------ENALLWTLNSLSMPNMDDFIEE***C***LREHNAKRELHGA------------

>F. hepatica c1795 ----------------------LLTMAGMDDFIEE***C***LREHNAKRSLHNA------------

>S. haematobium cA08278 -----MQQHLIFSFYTIFFLYKFILAIELTKDLDYLLKAHNQIRQDARD--***C***NRD***C***NITGQ

>C. elegans VAP1 -------------------------------------------------------SGGKNVYELNWD

>S. mansoni Smp012350.1 ---------------------------------------------------------------LRHS

>S. mansoni Smp012350.2 ---------------------------------------------------------------LRHS

>S. japonicum GAPR-1 ---------------------------------------------------------------LRHS

>F. gigantica c6239 --------------------------------------------------------------PLKFD

>F. hepatica c10262 --------------------------------------------------------------PLKFD

>S. haematobium cA07851 -------------------------------------------------------PPAKYMSPLKWN

>S. haematobium cA00818 -------------------------------------------------------PPAKYMSPLKWN

>C. sinensis c4469 -------------------------------------------------------------PALKHS

>O. viverrini c4948 -------------------------------------------------------------PALKHS

>F. hepatica c1795 -------------------------------------------------------------PALRHS

>S. haematobium cA08278 ITGQPQAKRLPNLMQQHLIFSFYTIFFLYKFILAIELTKDLDYLLKAHNQIRQDAPQAKRLPNLIWD

1 2
>C. elegans VAP1 ***C***EMEAKAQEWADG***C***PSSFQTFD-----------------------------------------------------------------

>S. mansoni Smp012350.1 LALDKTAQDWAEALLSEDGIKNSPLSSR-----------------------------------------------------------

>S. mansoni Smp012350.2 LALDKTAQDWAEALLSEDGIKNSPLSSR-----------------------------------------------------------

>S. japonicum GAPR-1 RALDKTAQDWAEELISDDGIKNSPLSSR-----------------------------------------------------------

>F. gigantica c6239 SELASQARMHAEDVIGQTQFEHEQ---------------------------------------------------------------

>F. hepatica c10262 SELASQARMHAEDVIGQTQFEHEQ---------------------------------------------------------------

>S. haematobium cA07851 YNLAAQAQTLANK***C***ILQHDKRHSDEFS------------------------------------------------------------

>S. haematobium cA00818 YDLAAQAQKLANQ***C***MFQQEIPYSDEFL------------------------------------------------------------

>C. sinensis c4469 RALDKTAQDWAEALISEPSIKNSPLSSR-----------------------------------------------------------

>O. viverrini c4948 RALDKTAQDWAEALISEPAIKNSPLSSR-----------------------------------------------------------

>F. hepatica c1795 RALDKTAQDWAEQLISEEQIKNSSLSGR-----------------------------------------------------------

>S. haematobium cA08278 NELASKAAALSKT***C***NFRFSNVTTKKFKDVGQNIAAYASVEIYGIMNWLQKRLHYRKRVIFVSPMQLRNLKMLDKIQLMLVLKLLMNG

3 2 3
>C. elegans VAP1 PTWGQNYATYMGSIADPLPYASMAVNGWWSEIRTVGLTDPDNKYTNSAMFRFANMANGKASAFG***C***AYAL***C***AGK-------LSIN***C***I

>S. mansoni Smp012350.1 GEVGESISVRTSTGTHVDMQGHEVVNTWHSDAENYNYENGKGPAGNFTQLVWSS-----TREVGFGKA***C***GPGK------***C***VVVAHY

>S. mansoni Smp012350.2 GEVGESISVRTSTGTHVDMQGHEVVNTWHSDAENYNYENGKGPAGNFTQLVWSS-----TREVGFGKA***C***GPGK------***C***VVVAHY

>S. japonicum GAPR-1 GEVGESISVRTSTGTHVDMQGHEVVNQWYSDAKNYNYENGKGPAGNFTQLVWSS-----TREVGFGKARGPGK------***C***VVVAHY

>F. gigantica c6239 SNDYGENLALRTGKEK***C***ILTGKQATLLWYSEIADYDFDQENQLSCGHFSQIVWKS----TTHAGFGKARSSDGS----KTVVVGVY

>F. hepatica c10262 SNDYGENLALRTGKEK***C***ILTGKQATLLWYSEIADYDFDQENQLSCGHFSQIVWKS----TTHAGFGKARSSDGSK----TVVVGVY

>S. haematobium cA07851 -------WVGQNIALHPT--------------------------------------------------------------------

>S. haematobium cA00818 ---***C***VGQNIVV***C***PT------IKSGVDAWFNEHKLYDYNQNN***C***ME***C***LHYTQMVWAK----TTDIG***C***GVAS***C***PKYG-----LSIV***C***NY

>C. sinensis c4469 GEVGESISMRTSSASHVDIQGNEVVNQWYADIKNYNFAEGKGPAGNFTQLVWKA-----TREVGFGKARSSGK------***C***IVVAHY

>O. viverrini c4948 GEVGESVSMRTSSASHVDIQGNEVVNQWYADIKNYNFTEGKGPAGNFTQLVWKA-----TREVGFGKARSSGK------***C***IVVAHY

>F. hepatica c1795 GEVGESISMRTSTASHVDIQGTEVVNQWYADMKNYNFESEK-GPAGNFTQLVWSA----TREVGFGKARAPGK------***C***IVVAHY

>S. haematobium cA08278 LMNITIIILIIIHVQLHVEIMYSAMNEWINEHNHYNFDNNT***C***TTS***C***GNYVQIVWHG---TTHIG***C***GVTY***C***PKTRRFPYGVFVV***C***NY

>C. elegans VAP1 -----------------------------------------------------------

>S. mansoni Smp012350.1 -----------------------------------------------------------

>S. mansoni Smp012350.2 -----------------------------------------------------------

>S. japonicum GAPR-1 -----------------------------------------------------------

>F. gigantica c6239 MPPANFENEWCENVPPPLSGQLYLPSKKEI***C***GTPEQHKKVIEKEKPENHTPEEKVIIIT

>F. hepatica c10262 MPPANFENEW***C***ENVPPPLSGQLYLPSKKEI***C***GTPEQHKKVIEKEKSENHAPEEKVIIIT-------

>S. haematobium cA07851 -----------------------------------------------------------

>S. haematobium cA00818 -----------------------------------------------------------

>C. sinensis c4469 -----------------------------------------------------------

>O. viverrini c4948 -----------------------------------------------------------

>F. hepatica c1795 -----------------------------------------------------------

>S. haematobium cA08278 -----------------------------------------------------------

>C. elegans VAP1 YNKIGYMTNAIIYEKGDA--------------------------------------------------------

>S. mansoni Smp012350.1 RPPGNVLGRYLENVFRPKKSVKDVKQPIRNTFALNN-DTPKTVITETLTESDGKQYSVRREISDLTDDKGKTRR

>S. mansoni Smp012350.2 RPPGNVLGRYLENVFRPKKSVKDVKQPIRNTFALNN-DTPKTVITETLTESDGKQYSVRREISDLTDDKGKTRR

>S. japonicum GAPR-1 RPPGNVLGRYLENVFRPKESTEESVEQLESNIFAFDSDIPKTVITETLTESDGKLYNVRREISESTDEKGKTRR

>F. gigantica c6239 RPTENHSSERNRQKIILVRENASKRTTTNSNRNTVIEQTTEIVSLQPSEELN----------------------

>F. hepatica c10262 RPTENHSSERNRQKIILVRENASNRTTTNSNRNTVIEQTTEIVSLQPSEELN----------------------

>S. haematobium cA07851 IKSGNWNNEKPYEVKSREL-------------------------------------------------------

>S. haematobium cA00818 GPGGNWTDEKPYEVKSREL-------------------------------------------------------

>C. sinensis c4469 RPPGNVRGHYAENVGTPTGEQAASVASATDTGNLDPNAKR-TVVTEEVTSPEGKRYTVHREVIETTEPDGHVRR

>O. viverrini c4948 RPPGNVRGHYAENVGAPTGGEAASVASATDTAKLDPDAKR-TVVTEEVTSPEGKRYTVHREVIETTEPDGHVRR

>F. hepatica c1795 RPPGNVRGHYAENVHPPTGGLPSGGGHSPTGSVQHAPGTKKTVVTENVTDPDGSKYTVRREVVESVGPDGRVKR

>S. haematobium cA08278 APGLYGMEQHTLDVVLLIVQKLDGSHMV----------------------------------------------

4 5 4 5
>C. elegans VAP1 ***C***TSDAE***C***TTYSDSQ---------***C***KNGL---------***C***YKAPQAPVVE--

>S. mansoni Smp012350.1 ***C***INEVYTDA--------------***C***KEQKKAKSSISPDGHLV---------

>S. mansoni Smp012350.2 ***C***INEVYTDA--------------***C***KEQKKAKSSISPDGHLV---------

>S. japonicum GAPR-1 ***C***VNEVYTDAYKKKGTTAPDVSQD***C***HSAD---------***C***S-----------

>F. gigantica c6239 ***C***TKAAKVTEIIQNG---------***C***ESDTFEVQTWYDVVTGTVIEDIGK--

>F. hepatica c10262 ***C***TKVAKVTEIIQKG---------***C***ESDTFEVQTWYDVVTGTVIKNFGK--

>S. haematobium cA07851 ***C***PKMQNIPKESFQTTRDDTQRAPSSIVNVNHKLSSIGSSEGNVNDQPSTG

>S. haematobium cA00818 ***C***PEKQNIPKDSFQTMRENTQRAPLSIANV--------------------- ... other (non PRP) domain (potential similarity to 1xta, 1rc9)

>C. sinensis c4469 ***C***VNETFQDSPDQATAG----------------------------------

>O. viverrini c4948 ***C***VNETFQDSPDQATAG----------------------------------

>F. hepatica c1795 VTSESFAEASDTITTESSTGPEAGGL------------------------

>S. haematobium cA08278 -------------------------------------------------- ... other (non PRP) domain (no hit found)

**Domain 2**

1

>C. elegans VAP1 TFTM***C***PSVTDQSDQARQNFLDTHNKLRTSLAKGLEADGIAAGAFAPMAKQMPK

>S. mansoni Smp012350.1 ------DNSKKLQESIHSVVQLHNQYRSQHGSNPL------------------

>S. mansoni Smp012350.2 ------DNSKKLQESIHSVVQLHNQYRSQHGSNPL------------------

>S. japonicum GAPR-1 ---------KPRQESIHSIVQMHNQYRSQHSSSPL------------------

>F. gigantica c6239 LAILRPIKSEQLLLEAFQIEVLHTQNRYRESHGVP------------------

>F. hepatica c10262 LAILRPIKSEQLLLEAFQIEVLHTQNRYRESHGVP------------------

>F. hepatica c1795 ---VTGNSKKDLESFGEAVTQVHNTHRARHGAP--------------------

>S. haematobium cA07851 -------LQTTLDDKSLEILELHRKYRQDLVNCKVDGQPPAKYMS--------
>C. sinensis c4469 GKHGASSEAAHGENFADAVTRAHNVYRKRHGVA--------------------

>O. viverrini c4948 DKHGAGSEAAHEENFADAVTRAHNVYRKRHGAA--------------------

1 2

>C. elegans VAP1 LVKYS***C***TVEANARTWAKG***C***LYQHSTSAQ------

>S. mansoni Smp012350.1 VLDQNLSNMAQQWADHLLQQSHLSNSGYVYRG--

>S. mansoni Smp012350.2 VLDQNLSNMAQQWADHLLQQSHLSNSGYVYRG--

>S. japonicum GAPR-1 VLDQNLSNMAQQWADHLLQQSHLSNSGFIYRG--

>F. gigantica c6239 SLKKNRELDRLAQSWAEELYNAEKLSYSNWEYNS

>F. hepatica c10262 SLKKNRELDRLAQSWAEELYNAEKLSYSNWEYNS

>F. hepatica c1795 ALKYDPQLSELAQKWAEELVQMPRLSNSGYTFEG

>S. haematobium cA07851 PLKWNYNLAAQAQTLANK***C***ILQHDKRH-------

>C. sinensis c4469 DLQLDPEISHMAQDWAEQLVNRAHLSNSGFTYQG

>O. viverrini c4948 DLQLDPEISHMAQDWAEQLVNRAHLSNSGFTYQG

>C. elegans VAP1 RPGLGENLYMISINNMPK-IQTAEDSSKAWWSELKDFGVGSDNILTQAVFDRGVGHY
>S. mansoni Smp012350.1 --MKVGENLGSRWSNGPM-ELNCKDLIEHWYQESGKYKFNSEPDSIQGIGNFTQIVW

>S. mansoni Smp012350.2 --MKVGENLGSRWSNGPM-ELNCKDLIEHWYQESGKYKFNSEPDSIQGIGNFTQIVW

>S. japonicum GAPR-1 --LKLGENVGSRWSNGSI-ELNCKELIEHWYQESEKYNFDSEPDSIQGIGNFTQIVW

>F. gigantica c6239 RP--LGE-IVS***C***RTWKNS-RISGEVLVSKWYHESQSYDYTREPDSSTRSGWFTQLIW

>F. hepatica c10262 RP--LGE-IVS***C***RTRKNS-RISGEVLVSKWYHESQSYDYTREPDSSTRSGWFTQLIW

>F. hepatica c1795 VR--LGENVLSRWSTAAV-HFGAQDLVDHWYQECSKYKFDTEPSSIQGIGGFTQVVW

>S. haematobium cA07851 -----SDEFSWVGQNIAL-HPTIKSGVDAWFNEHKLYDY------------------

>C. sinensis c4469 VR--LGENVL-***C***RWSNTAATVSAQDVVDHWYQESSKYKFTSEPKSIQGIGGFTQVVW

>O. viverrini c4948 VR--LGENVL-***C***RWSNTTATVSAQDVVDHWYQESSKYNFNSEPKSIQGIGGFTQMVW

3 2 3 4

>C. elegans VAP1 TQMAWEGTTEIG***C***FVEN---------***C***P-TFTYSV***C***QYGPAGNYMNQLIYTKGSP***C***TADAD----------------
>S. mansoni Smp012350.1 ----SSSERIGVGIAIQSYKSGEDLHKD-SKMILV***C***LYHPPGNVISQFQNNVKKAIK--------------------

>S. mansoni Smp012350.2 ----SSSERIGVGIAIQSYKSGEDLHKD-SKMILV***C***LYHPPGNVISQFQNNVKKAIK--------------------

>S. japonicum GAPR-1 ----SNSEIIGVGIASQSYETGEALRKD-SKLILV***C***LYYPPGNVISQFKNNVKKAIKINTGA---------------

>F. gigantica c6239 ---KDSRELGVGFIPSR---------EP-DSVIIV***C***FYFPTGNVKGKFVDNVLPPIMDDDVNGTKNGQL--------

>F. hepatica c10262 ---KDSRELGVGFIPSR---------EP-DSVIIV***C***FYFPTGNVKGKFVDNVLPPIMDDDVNGTKEGQLISFSQIFV

>F. hepatica c1795 ---SGSQRIGVGRAMQPKSGPGGSTSGPGYKMVAV***C***FYYPPGNVTGQFKTNVKPSSRYFPNRWPYITSRYMELPHL-

>S. haematobium cA07851 ----------------------NHND***C***MK------***C***LHY--------------------------------------

>C. sinensis c4469 ---NGSQRIGVGIASQAKKDFYNQPSQSKVIVV***C***FYYPPGNVTGQFRANVKQGMNLKSSAPVKYCHPFSMKAGLSFR

>O. viverrini c4948 ---SGSQRIGVGIASQPKKDFYNQPSQSKVIVV***C***FYYPPGNVTGQFRANVKQGMNLISSTPVKYCHQFSIKADFSSI

>C. elegans VAP1 -----------------------------------------------------------------------------

>S. mansoni Smp012350.1 -----------------------------------------------------------------------------

>S. mansoni Smp012350.2 -----------------------------------------------------------------------------

>S. japonicum GAPR-1 -----------------------------------------------------------------------------

>F. gigantica c6239 -----------------------------------------------------------------------------

>F. hepatica c10262 -----------------------------------------------------------------------------

>F. hepatica c1795 -----------------------------------------------------------------------------

>S. haematobium cA07851 TQNIPKESFRTTRYDTQ--------RAP-SSMLNVNHKLLMTGSSRSRVHHQRTHDQPISINTFQYSRRNTQRKRIS

>C. sinensis c4469 -----------------------------------------------------------------------------

>O. viverrini c4948 -----------------------------------------------------------------------------

5 4 5

>C. elegans VAP1 ***C***PGTQT***C***SVAEAL***C***VIP--------------------------------------------------------------

>S. mansoni Smp012350.1 -------------------------------------------------------------------------------

>S. mansoni Smp012350.2 -------------------------------------------------------------------------------

>S. japonicum GAPR-1 -------------------------------------------------------------------------------

>F. gigantica c6239 -------------------------------------------------------------------------------

>F. hepatica c10262 IKNLFSVLVLEWN***C***NKIEINIRRTMNDFX--------------------------------------------------

>F. hepatica c1795 ***C***FISLFIAVSVPLIHINDEHLHLHILRNSIHNFVWSVSILFIYLCIQPSFPPRSHWFYTMFTCLRPHLRTCTLLAFPPP

>S. haematobium cA07851 GKKALDKFSKSMRGRIGHGNVPRQQNIRIVRYRN---------------------------------------------

>C. sinensis c4469 IRPLVKYDYRTPTLTDIYAALTFALLPDTSYKLILFLHFSFLSGKAYLFGSLLYPQNTLFQR-----------------
>O. viverrini c4948 IRPLVKYEYRTPTLTDIHAALTFASRHFLQVNYLFAX------------------------------------------

**non-PRP domains (N-terminal)**

>C. sinensis c4469 TGRQSYIFALGTSSQKCIQCTWYSRNSMEIILTTHSEAFSPIVSIFELCDIWTYVIPTISRWNVPCFHKEQRPKVIFLLESRTVFSMAVNGDVVVLIKTSVH

**non-PRP domains (C-terminal)**

>S. haematobium cA00818 NHKLSSTGSSGSRVHERRPNSQCVPTNIIQNNLFNTKSIYGKKALHMLLKS
>S. haematobium cA00818 VRSGSGLGYVQGQHNNRRLVWQNTTHVGCGITNCTGNYGFPYGLSVVCNYG
>S. haematobium cA00818 PGGNYEGRYPYEAKSQDECYAVTTRRPKTTRRPITTKRPKTTRRPGTIPTR
>S. haematobium cA00818 KPDISKQIPKPNWPTLISSWSGFANSNMLHGIVTKTCICIQ

>S. haematobium cA08278 YLYVTTLLEQSLIEVHMMSAMQNVQLVNGNGNSLIMEK
>S. haematobium cA08278 IVIVIYEAKFDRSPYDVVSYAKCPTGQWKWKQFNLNGENCYCYL
