# Supplementary material for: Insights into SCP/TAPS Proteins of Liver Flukes Based on Large-Scale Bioinformatic Analyses of Sequence Datasets
Source: PLoS One. 2012 Feb 22;7(2):e31164. doi: 10.1371/journal.pone.0031164 (PMC3284463; doi:10.1371/journal.pone.0031164)
Supplement: Table S1 — A summary of the characteristics of putative single-domain SCP/TAPS predicted from the transcriptomic datasets from Clonorchis sinensis , Opisthorchis viverrini , Fasciola hepatica and F. gigantica (sequence data is available for download from http://www.gasserlab.org/ ) and from the genomic datasets from Schistosoma mansoni , S. japonicum and S. haematobium . (DOC) [file pone.0031164.s003.doc]

Table S1

|  | Contig name | Length of predicted protein (amino acids) | Best amino acid sequence match (e-value) | Signal peptide | Structural  Group |
| --- | --- | --- | --- | --- | --- |
| *Clonorchis sinensis* |  |  |  |  |  |
|  | c10021 | 37 | PR-1 homolog [Solanum melongena] (1e-06) | No | 2 |
|  | c1246 | 333 | venom allergen-like (VAL) 26 protein [Schistosoma mansoni] (1e-44) | Yes | 1 |
|  | c1247 | 335 | venom allergen-like protein 8 [Schistosoma mansoni] (4e-30) | No | 1 |
|  | c13355 | 129 | cysteine-rich protease inhibitor [Mus musculus] (1e-12) | No | 2 |
|  | c13576 | 166 | venom allergen-like (VAL) 13 protein [Schistosoma mansoni] (3e-27) | No | 3 |
|  | c14383 | 128 | SJCHGC09193 protein [Schistosoma japonicum] (9e-16) | Yes | 2 |
|  | c14468 | 56 | GLI pathogenesis-related 2 [Xenopus (Silurana) tropicalis] (1e-07) | No | 3 |
|  | c1770 | 268 | venom allergen-like (VAL) 13 protein [Schistosoma mansoni] (6e-22) | No | 3 |
|  | c2042 | 139 | Golgi-associated plant pathogenesis-related protein 1 [Schistosoma japonicum] (1e-12) | No | 3 |
|  | c2194 | 279 | venom allergen-like (VAL) 8 protein [Schistosoma mansoni] (1e-21) | Yes | 1 |
|  | c3048 | 205 | SCP-like family protein [Plesiocystis pacifica SIR-1] (7e-11) | No | 2 |
|  | c3166 | 206 | SCP-like extracellular protein [Schistosoma japonicum] (8e-46) | No | 3 |
|  | c353 | 240 | venom allergen-like (VAL) 27 protein [Schistosoma mansoni] (2e-32) | Yes | 1 |
|  | c3765 | 338 | venom allergen-like protein 13 [Clonorchis sinensis] (2e-73) | No | 3 |
|  | c4257 | 239 | venom allergen-like protein [Clonorchis sinensis] (6e-108) | Yes | 1 |
|  | c4741 | 330 | extracellular SCP domain-containing protein Pry1 [Metarhizium anisopliae] (6e-05) | No | 2 |
|  | c5350 | 215 | PREDICTED: peptidase inhibitor 16-like [Sus scrofa] (1e-12) | No | 2 |
|  | c6993 | 58 | venom allergen-like (VAL) 16 protein [Schistosoma mansoni] (2e-10) | No | 3 |
|  | c7351 | 324 | glioma pathogenesis-related protein 1-like [Monodelphis domestica] (1e-08) | Yes | 2 |
|  | c8455 | 202 | venom allergen-like (VAL) 16 protein [Schistosoma mansoni] (1e-05) | No | 3 |
| *Opisthorchis viverrini* |  |  |  |  |  |
|  | c11437 | 279 | SCP-like extracellular protein, putative [Microcoleus chthonoplastes PCC 7420] (1e-18) | No | 3 |
|  | c1174 | 221 | GLIPR1-like protein 1 precursor [Schistosoma japonicum] (7e-31) | No | 1 |
|  | c11939 | 162 | SJCHGC08973 protein [Schistosoma japonicum] (1e-07) | No | 3 |
|  | c15215 | 66 | GLIPR1-like protein 1 precursor [Schistosoma japonicum] (2e-03) | No | 1 |
|  | c15680 | 84 | hypothetical protein CaO19.9872 [Candida albicans SC5314] (1e-15) | No | 2 |
|  | c17638 | 61 | venom allergen-like (VAL) 27 protein [Schistosoma mansoni] (1e-09) | No | 2 |
|  | c18119^¤^ | 118 | pathogenesis-related protein 1-23 [Triticum aestivum] (2e-16) | No | 2 |
|  | c18160^¤^ | 107 | pathogenesis-related protein 1 [Musa ABB Group] (1e-15) | No | 2 |
|  | c18440 | 286 | venom allergen-like (VAL) 13 protein [Schistosoma mansoni] (1e-53) | No | 3 |
|  | c19644^¤^ | 78 | venom allergen-like protein 8 [Schistosoma mansoni] (1e-15) | Yes | 2 |
|  | c19841 | 46 | activation-associated secreted protein-1 [Onchocerca volvulus] (3e-04) | No | 4 |
|  | c2349^§^ | 411 | venom allergen-like protein 13 [Clonorchis sinensis] (1e-41) | No | 3 |
|  | c2686^§^ | 264 | GLIPR1-like protein 1 precursor [Schistosoma japonicum] (7e-58) | Yes | 1 |
|  | c3648 | 131 | PREDICTED: peptidase inhibitor 16-like [Equus caballus] (5e-08) | No | 2 |
|  | c3766^§^ | 237 | venom allergen-like (VAL) 13 protein [Schistosoma mansoni] (9e-25) | No | 3 |
|  | c4326 | 191 | venom allergen-like protein [Clonorchis sinensis] (7e-96) | No | 1 |
|  | c4390^§^ | 299 | SCP-like extracellular protein [Schistosoma japonicum] (2e-58) | No | 3 |
|  | c4477 | 251 | extracellular SCP domain-containing protein Pry1 [Metarhizium anisopliae ARSEF 23] (9e-08) | No | 2 |
|  | c5997 | 107 | SCP-related protein [Bombyx mori] (7e-12) | No | 2 |
|  | c6176 | 204 | SJCHGC08973 protein [Schistosoma japonicum] (6e-44) | No | 3 |
|  | c6515 | 350 | venom allergen-like protein 8 [Schistosoma mansoni] (5e-35) | No | 1 |
|  | c7991 | 166 | GLIPR1-like protein 1 precursor [Schistosoma japonicum] (2e-36) | Yes | 2 |
|  | c8913 | 203 | venom allergen-like protein 13 [Clonorchis sinensis] (1e-82) | No | 3 |
|  | c962 | 283 | venom allergen-like protein 8 [Schistosoma mansoni] (9e-35) | No | 1 |
| *Fasciola hepatica* |  |  |  |  |  |
|  | c10643^¤^ | 355 | venom allergen-like (VAL) 13 protein [Schistosoma mansoni] (6e-43) | No | 3 |
|  | c1472 | 298 | SJCHGC08973 protein [Schistosoma japonicum] (2e-40) | No | 3 |
|  | c4375^¤^ | 332 | GLIPR1-like protein 1 precursor [Schistosoma japonicum] (3e-45) | No | 1 |
|  | c6081 | 229 | Cysteine-rich secretory protein LCCL domain-containing 2 precursor [Osmerus mordax] (5e-20) | No | 2 |
|  | c6577 | 274 | Glioma pathogenesis-related protein 1 precursor [Schistosoma japonicum] (6e-30) | Yes | 1 |
|  | c8830 | 96 | venom allergen-like protein 8 [Schistosoma mansoni] (2e-16) | No | 1 |
|  | c8831^¤^ | 109 | venom allergen-like (VAL) 8 protein [Schistosoma mansoni] (1e-06) | No | 1 |
|  | c9115 | 129 | Golgi-associated plant pathogenesis-related protein 1 [Schistosoma japonicum] (3e-09) | No | 3 |
|  | c9426^¤^ | 451 | SJCHGC08973 protein [Schistosoma japonicum] (2e-51) | No | 3 |
| *Fasciola gigantica* |  |  |  |  |  |
|  | c1042 | 202 | SJCHGC08973 protein [Schistosoma japonicum] (5e-41) | No | 3 |
|  | c12544 | 197 | SJCHGC09417 protein [Schistosoma japonicum] (5e-82) | No | 3 |
|  | c12861 | 221 | venom allergen-like protein 13 [Clonorchis sinensis] (8e-37) | No | 3 |
|  | c25411 | 99 | SJCHGC09193 protein [Schistosoma japonicum] (4e-21) | No | 2 |
|  | c3823 | 240 | venom allergen-like (VAL) 16 protein [Schistosoma mansoni] (1e-57) | No | 3 |
|  | c3 | 255 | venom allergen-like protein 13 [Schistosoma mansoni] (3e-40) | No | 3 |
|  | c4035 | 259 | Golgi-associated plant pathogenesis-related protein 1 [Schistosoma japonicum] (1e-32) | No | 3 |
|  | c4654 | 412 | venom allergen-like (VAL) 11 protein [Schistosoma mansoni] (1e-54) | No | 3 |
|  | c566 | 254 | venom allergen-like protein 8 [Schistosoma mansoni] (2e-39) | No | 1 |
|  | c7108 | 209 | Glioma pathogenesis-related protein 1 precursor [Schistosoma japonicum] (7e-31) | No | 1 |
| *Schistosoma haematobium* |  |  |  |  |  |
|  | cA00413 | 99 | venom allergen-like protein 10 [Schistosoma mansoni] (7e-40) | No | 4 |
|  | cA00481 | 189 | venom allergen-like (VAL) 13 protein [Schistosoma mansoni] (2e-109) | No | 3 |
|  | cA00727 | 180 | SJCHGC08640 protein [Schistosoma japonicum] (4e-44) | No | 3 |
|  | cA01429 | 125 | venom allergen-like (VAL) 4 protein [Schistosoma mansoni] (4e-57) | Yes | 1 |
|  | cA01987 | 137 | venom allergen-like (VAL) 17 protein [Schistosoma mansoni] (1e-83) | No | 3 |
|  | cA03186 | 186 | SJCHGC06310 protein [Schistosoma japonicum] (1e-98) | No | 3 |
|  | cA03835 | 178 | hypothetical protein [Schistosoma mansoni] (1e-73) | No | 4 |
|  | cA06111 | 70 | venom allergen-like (VAL) 9 protein [Schistosoma mansoni] (8e-14) | No | 2 |
|  | cA06447 | 195 | venom allergen-like (VAL) 18 protein [Schistosoma mansoni] (5e-111) | No | 1 |
|  | cA06474 | 231 | venom allergen-like (VAL) 20 protein [Schistosoma mansoni] (2e-113) | No | 1 |
|  | cA06788 | 171 | venom allergen-like (VAL) 6 protein [Schistosoma mansoni] (5e-105) | No | 4 |
|  | cA07187 | 103 | venom allergen-like (VAL) 11 protein [Schistosoma mansoni] (3e-42) | No | 4 |
|  | cA00818 | 399 | venom allergen-like (VAL) 2 protein [Schistosoma mansoni] (4e-86) | Yes | - |
|  | cA08278 | 408 | venom allergen-like (VAL) protein 12 [Schistosoma mansoni] (1e-15) | Yes | - |
|  | cA07983 | 121 | unknown [Schistosoma japonicum] (3e-67) | No | 3 |
|  | cA08206 | 227 | venom allergen-like (VAL) 7 protein [Schistosoma mansoni] (2e-61) | No | 2 |
|  | cA08594 | 200 | venom allergen-like (VAL) 24 protein [Schistosoma mansoni] (1e-102) | Yes | 1 |
|  | cA08595 | 185 | venom allergen-like (VAL) 24 protein [Schistosoma mansoni] (2e-98) | Yes | 1 |
|  | cA08596 | 185 | venom allergen-like (VAL) 24 protein [Schistosoma mansoni] (2e-98) | Yes | 1 |
|  | cA08597 | 209 | venom allergen-like (VAL) 24 protein [Schistosoma mansoni] (1e-98) | Yes | 2 |
|  | cA08598 | 264 | venom allergen-like protein 5 [Schistosoma mansoni] (3e-148) | Yes | 2 |
|  | cA09053 | 181 | venom allergen-like (VAL) 26 protein [Schistosoma mansoni] (1e-110) | Yes | 2 |
|  | cB00359 | 150 | venom allergen-like (VAL) 11 protein [Schistosoma mansoni] (1e-96) | No | 3 |
|  | cC00745 | 140 | crisp subfamily glioma pathogenesis-related protein-related [Schistosoma mansoni] (2e-79) | No | 2 |
| *Schistosoma japonicum* |  |  |  |  |  |
|  | Allergen V5/Tpx-1 (1) | 67 | unknown [Schistosoma japonicum] (2^e-28^) | No | 2 |
|  | GAPR-1 (1) | 386 | SJCHGC08973 protein [Schistosoma japonicum] (3e-66) | No | 3 |
|  | GAPR-1 (2) | 131 | SJCHGC06310 protein [Schistosoma japonicum] (6e-88) | No | 3 |
|  | GLIPR-1 (1) | 184 | GLIPR1-like protein 1 precursor [Schistosoma japonicum] (1e-133) | Yes | 2 |
|  | GLIPR-1 (2) | 230 | venom allergen-like protein 1 [Schistosoma mansoni] (2e-84) | No | 1 |
|  | Peptidase inhibitor 16 (1) | 274 | venom allergen-like protein 1 [Schistosoma mansoni] (2e-84) | No | 1 |
|  | GLIPR-1 (3) | 242 | venom allergen-like protein 8 [Schistosoma mansoni] (2e-109) | Yes | 1 |
|  | GLIPR-1 (4) | 205 | Glioma pathogenesis-related protein 1 precursor [Schistosoma japonicum] (8e-54) | Yes | 1 |
|  | Peptidase inhibitor 16 (2) | 191 | SJCHGC01839 protein [Schistosoma japonicum] (2e-134) | No | 2 |
|  | Peptidase inhibitor 16 (3) | 215 | hypothetical protein [Schistosoma japonicum] (4e-122) | Yes | 1 |
|  | Allergen V5/Tpx-1 (2) | 173 | venom allergen-like (VAL) 20 protein [Schistosoma mansoni] (2e-62) | No | 2 |
|  | Allergen V5/Tpx-1 (3) | 353 | SJCHGC09193 protein [Schistosoma japonicum] (4e-120) | No | 4 |
|  | Expressed protein | 125 | venom allergen-like (VAL) 4 protein [Schistosoma mansoni] (6e-43) | Yes | 1 |
|  | GAPR-1 (3) | 159 | Golgi-associated plant pathogenesis-related protein 1 [Schistosoma japonicum] (1e-117) | No | 2 |
|  | GLIPR-1 (5) | 204 | GLIPR1-like protein 1 precursor [Schistosoma japonicum] (3e-153) | No | 2 |
|  | PRP-4 | 221 | SJCHGC06484 protein [Schistosoma japonicum] (6e-165) | Yes | 2 |
| *Schistosoma mansoni* |  |  |  |  |  |
|  | Smp_001890 | 194 | venom allergen-like (VAL) 16 protein [Schistosoma mansoni] (1e-144) | No | 1 |
|  | Smp_002060 | 170 | venom allergen-like (VAL) 10 protein [Schistosoma mansoni] (9e-124) | Yes | 2 |
|  | Smp_002070 | 181 | venom allergen-like (VAL) 4 protein [Schistosoma mansoni] (3e-132) | Yes | 1 |
|  | Smp_002630 | 229 | venom allergen-like (VAL) 2 protein [Schistosoma mansoni] (6e-173) | No | 1 |
|  | Smp_035980 | 264 | histone H2A [Schistosoma mansoni] (0.0) | Yes | 1 |
|  | Smp_070240 | 193 | venom allergen-like (VAL) 7 protein [Schistosoma mansoni] (5e-146) | No | 2 |
|  | Smp_070250 | 270 | venom allergen-like (VAL) 15 protein [Schistosoma mansoni] (0.0) | No | 2 |
|  | Smp_078490 | 219 | venom allergen-like (VAL) 14 protein [Schistosoma mansoni] (5e-165) | Yes | 1 |
|  | Smp_100560 | 100 | hypothetical protein [Schistosoma mansoni] (3e-70) | No | 1 |
|  | Smp_116210 | 100 | hypothetical protein [Schistosoma mansoni] (9e-71) | No | 1 |
|  | Smp_118160 | 100 | hypothetical protein [Schistosoma mansoni] (3e-69) | No | 1 |
|  | Smp_120240 | 100 | venom allergen-like (VAL) 1 protein [Schistosoma mansoni] (2e-69) | No | 1 |
|  | Smp_120670 | 141 | crisp subfamily glioma pathogenesis-related protein-related [Schistosoma mansoni] (3e-140) | No | 2 |
|  | Smp_123090 | 187 | venom allergen-like (VAL) 19 protein [Schistosoma mansoni] (6e-139) | Yes | 1 |
|  | Smp_123540 | 204 | venom allergen-like (VAL) protein 12 [Schistosoma mansoni] (2e-152) | No | 1 |
|  | Smp_123550 | 262 | venom allergen-like protein 8 [Schistosoma mansoni] (0.0) | No | 1 |
|  | Smp_124050.1 | 360 | venom allergen-like (VAL) 6 protein [Schistosoma mansoni] (0.0) | No | 3 |
|  | Smp_124050.2 | 360 | venom allergen-like (VAL) 6 protein [Schistosoma mansoni] (0.0) | No | 3 |
|  | Smp_124050.3 | 360 | venom allergen-like (VAL) 6 protein [Schistosoma mansoni] (0.0) | No | 3 |
|  | Smp_124050.4 | 360 | venom allergen-like (VAL) 6 protein [Schistosoma mansoni] (0.0) | No | 3 |
|  | Smp_124060 | 236 | venom allergen-like (VAL) 13 protein [Schistosoma mansoni] (1e-179) | No | 3 |
|  | Smp_124070 | 169 | venom allergen-like (VAL) 16 protein [Schistosoma mansoni] (4e-125) | No | 3 |
|  | Smp_127130 | 225 | venom allergen-like (VAL) 20 protein [Schistosoma mansoni] (5e-170) | No | 1 |
|  | Smp_131370 | 148 | hypothetical protein [Schistosoma mansoni] (2e-105) | No | 4 |
|  | Smp_139450 | 219 | venom allergen-like (VAL) 22 protein [Schistosoma mansoni] (1e-164) | Yes | 1 |
|  | Smp_141550 | 196 | venom allergen-like (VAL) 24 protein [Schistosoma mansoni] (7e-147) | Yes | 1 |
|  | Smp_141560 | 196 | venom allergen-like (VAL) 25 protein [Schistosoma mansoni] (3e-146) | Yes | 1 |
|  | Smp_154260 | 183 | venom allergen-like (VAL) 26 protein [Schistosoma mansoni] (7e-136) | Yes | 2 |
|  | Smp_154290 | 183 | venom allergen-like (VAL) 27 protein [Schistosoma mansoni] (2e-136) | Yes | 2 |
|  | Smp_159280 | 223 | crisp subfamily glioma pathogenesis-related protein-related [Schistosoma mansoni] (2e-163) | Yes | 1 |
|  | Smp_159290 | 235 | venom allergen-like (VAL) 21 protein [Schistosoma mansoni] (3e-178) | Yes | 1 |
|  | Smp_160250 | 201 | venom allergen-like (VAL) 23 protein [Schistosoma mansoni] (3e-151) | Yes | 1 |
|  | Smp_163400 | 169 | venom allergen-like (VAL) 17 protein [Schistosoma mansoni] (1e-125) | No | 3 |
|  | Smp_176160 | 183 | venom allergen-like (VAL) 28 protein [Schistosoma mansoni] (3e-136) | Yes | 2 |
|  | Smp_176170 | 88 | hypothetical protein [Schistosoma mansoni] (3e-58) | Yes | 2 |
|  | Smp_176180 | 182 | venom allergen-like (VAL) 9 protein [Schistosoma mansoni] (5e-137) | Yes | 2 |
|  | Smp_179480 | 270 | venom allergen-like (VAL) 5 protein [Schistosoma mansoni] (0.0) | No | 2 |
|  | Smp_193680 | 164 | venom allergen-like (VAL) 1 protein [Schistosoma mansoni] (4e-122) | No | 1 |
|  | Smp_193710 | 213 | venom allergen-like (VAL) 3 protein [Schistosoma mansoni] (3e-160) | Yes | 1 |

^¤^ Up-regulated in the juvenile stages of *Opisthorchis viverrini* and *Fasciola hepatica*

^§^ Up-regulated in the adult stage of *Opisthorchis viverrini*
