# Supplementary material for: Insights into SCP/TAPS Proteins of Liver Flukes Based on Large-Scale Bioinformatic Analyses of Sequence Datasets
Source: PLoS One. 2012 Feb 22;7(2):e31164. doi: 10.1371/journal.pone.0031164 (PMC3284463; doi:10.1371/journal.pone.0031164)
Supplement: Table S2 — A summary of the characteristics of putative double(SCP-extracellular)-domain SCP/TAPS predicted from the transcriptomic datasets from Clonorchis sinensis , Opisthorchis viverrini , Fasciola hepatica and F. gigantica (sequence data is available for download from http://www.gasserlab.org/ ) and from the genomic datasets from Schistosoma mansoni , S. japonicum and S. haematobium . (DOC) [file pone.0031164.s004.doc]

Table S2.

| Species | Contig name | Length of predicted protein (amino acids) | Best amino acid sequence match (e-value) | Signal peptide | Structural  group |
| --- | --- | --- | --- | --- | --- |
| *Clonorchis sinensis* |  |  |  |  |  |
|  | c4469 | 598 | SJCHGC09417 protein [Schistosoma japonicum] (4e-128) | No | 2 |
| *Opisthorchis viverrini* |  |  |  |  |  |
|  | c4948^¤^ | 469 | SJCHGC09417 protein [Schistosoma japonicum] (1e-131) | No | 2 |
| *Fasciola hepatica* |  |  |  |  |  |
|  | c10262^¤^ | 525 | Golgi-associated plant pathogenesis-related protein 1 [Schistosoma japonicum] (3e-38) | No | 2 |
|  | c1795^¤^ | 517 | SJCHGC09417 protein [Schistosoma japonicum] (3e-123) | No | 2 |
| *Fasciola gigantica* |  |  |  |  |  |
|  | c6239 | 459 | Golgi-associated plant pathogenesis-related protein 1 [Schistosoma japonicum] (1e-38) | No | 2 |
| *Schistosoma haematobium* |  |  |  |  |  |
|  | cA07851 | 365 | venom allergen-like protein 1 [Schistosoma mansoni] (6e-49) | No | 1 |
| *Schistosoma japonicum* |  |  |  |  |  |
|  | GAPR-1 | 406 | SJCHGC09417 protein [Schistosoma japonicum] (0.0) | No | 2 |
| *Schistosoma mansoni* |  |  |  |  |  |
|  | Smp_012350.1 | 400 | venom allergen-like (VAL) 11 protein [Schistosoma mansoni] (0.0) | No | 2 |
|  | Smp_012350.2 | 400 | venom allergen-like (VAL) 11 protein [Schistosoma mansoni] (0.0) | No | 2 |

^¤^ Up-regulated in the juvenile stages of *Opisthorchis viverrini* and *Fasciola hepatica*
